# Polymorphisms within the Telomerase Reverse Transcriptase gene (TERT) in four breeds of dogs selected for difference in lifespan and cancer susceptibility

**DOI:** 10.1186/1746-6148-10-20

**Published:** 2014-01-14

**Authors:** Camille A McAloney, Kevin A T Silverstein, Jaime F Modiano, Anindya Bagchi

**Affiliations:** 1 Augsburg College, Minneapolis, MN 55454, USA; 2 College of Veterinary Medicine, University of Minnesota, St. Paul, MN 55108, USA; 3 Masonic Cancer Center, University of Minnesota, Minneapolis, MN 55455, USA; 4 Department of Veterinary Clinical Sciences, College of Veterinary Medicine, University of Minnesota, St. Paul, MN 55108, USA; 5 Department of Genetics, Cell Biology and Development, School of Medicine, 420 Delaware St. SE MMC 806, Minneapolis, MN 55455, USA

## Abstract

**Background:**

Enzymatic activity of *Te*lomerase *R*everse *T*ranscriptase (TERT) is important in maintaining the telomere length and has been implicated in cancer and aging related pathology. Since cancer susceptibility as well as longevity of dogs vary between breeds, this study involved sequencing the entire *TERT* gene of *Canis familiaris* from DNA samples obtained from forty dogs, with ten dogs each of four breeds: Shih Tzu, Dachshund, Irish Wolfhound, and Newfoundland, each with different life expectancies and susceptibility to cancer.

**Results:**

We compared the sequences of all forty individuals amongst one another and with the published sequence of canine *TERT*, and analyzed relationships between members of the same or different breeds. Two separate phylogenetic trees were generated and analyzed from these individuals. Polymorphisms were found most frequently in intronic regions of the gene, although exonic polymorphisms also were observed. In many locations genotypes were observed that were either homozygous for the reference sequence or heterozygous, but the variant homozygous genotype was not observed.

**Conclusions:**

We propose that these homozygous variants are likely to have adverse effects in dogs. It was also found that the polymorphisms did not segregate by breed. Because the four breeds chosen come from geographically and physiologically distinct backgrounds, it can be inferred that the polymorphic diversification of *TERT* preceded breed derivation.

## Background

Telomeres are linked to susceptibility to cancer, the aging process, and the lifespan of individual cells [1]. Also, the dysfunction of a single enzyme involved in telomere maintenance has been shown to be sufficient to cause severe telomere damage, premature tissue aging, and development of neoplastic lesions [2]. Telomere length is controlled by the enzyme telomerase, a component of which is *Te*lomerase *R*everse *T*ranscriptase (*TERT)*. Therefore, variations in telomerase activity could result from coding polymorphisms affecting the enzymatic function of *TERT*, or from non-coding polymorphisms affecting the magnitude or kinetics of *TERT* expression. Interestingly, it has also been observed that while deficient telomerase activity results in a decreased rate of cancer in mice [3], over-expression and ubiquitious expression of telomerase result in an increased rate of tumors [4].

Domestic dogs provide an exceptional experimental model to assess the potential effect of such polymorphisms on organismal lifespan, as it is well recognized that many large breed dogs age faster and have shorter life spans than dogs of small breeds. Furthermore, cancer susceptibility varies widely by breed [5] making dogs excellent models for studying cancer susceptibility. It has been specifically shown that in dogs, as in humans, cancer cells rely on telomerase to maintain telomeres and extend their lifespan [6].

For this study, polymorphisms in the *TERT* sequence were observed in Shih Tzus, Dachshunds, Irish Wolfhounds and Newfoundlands: four breeds that show large differences in size, expected lifespan, cancer susceptibility, and geographic origins. Shih Tzus are small dogs that have an average lifespan of 13 years [7]; the breed is thought to be “ancient”, and it dates back at least to the year 624 A.D. when it was first depicted in Chinese art [8]. These dogs are thought to be at a decreased risk for cancer on the whole [5]. Dachshunds are small dogs that were first bred in Germany in the early 1600s [8] and also have an average lifespan of 13 years [7]. Evidence points to them having a decreased risk of cancer compared to the average, but they appear to have a predilection for mammary tumors [9]. Records of Irish Wolfhounds, large dogs first bred in Ireland, date back to 391 A.D. [8]. These dogs only live 7 years on average [7], and are particularly susceptible to osteosarcoma, even as compared to other large, fast-growing breeds that are typically prone to this cancer [10]. Finally, the large Newfoundland breed was developed in Newfoundland, Canda in the early 1600s [8] and live 9 years on average [7]. These dogs are thought to be no more or less susceptible to cancer than average [5].

Although reproductive isolation among breeds was not formally standardized until the mid-19th century with the formation of breed clubs and breed standards [11], geographic isolation of these breeds in their place of origin makes interbreeding among these breeds unlikely. Furthermore, Parker et al. showed that modern dog breeds are “distinct genetic units” and assigned the dog breeds used in this study to genetically distinct “clusters”.

Our objectives here were to assess conserved polymorphism in TERT, and to establish if they segregated according to the breeds or randomly. In order to do so, we sequenced the TERT gene of 10 dogs each from the aforementioned breeds and compared their sequences and any polymorphism that were found.

## Methods

### Samples

Dr. Heidi Parker (NHGRI, NIH, Bethesda, MD) provided de-identified and breed-verified samples from forty dogs including ten Shi Tzu, ten Dachshund, ten Newfoundland, and ten Irish Wolfhounds**.** All ethical approvals for protocols, including IACUC approval for collection of the samples, were obtained for this study.

### TERT sequence

We used the genomic sequence of canine *TERT* (TERT_CANFA ENSCAFT00000017081 CanFam 2.0) [Release 58 May 2010 [12]] as a reference, including 500 additional nucleotides flanking either end of the gene. The plasmid editing program, ApE (Wayne Davis; Salt Lake City, Utah; version “ApE_OSX_1_1_7.dmg”) [13] was used to parcel exons, introns, and previously catalogued polymorphisms. As depicted in Additional file 1: Figure S1, the gene was divided into 500 nucleotide-long segments, with each segment overlapping by 250 nucleotides, for a total of 74 sections. The second primer pair could not be generated due to extensive GC pairing. ApE was used to generate forward and reverse primers for each section. We then manually reviewed each list of potential primers per section, with the final primer pairs chosen based on stability, GC content, melting point, and salt content. The primer sequences are listed in Additional file 1: Table S1.

### DNA extraction

We extracted DNA from lymph node tissue of a healthy dog obtained with IACUC approval, and used it at a concentration of 40 ng/μL to standardize the conditions of PCR amplification. The performance of each pair was verified by conventional PCR and gel electrophoresis. Of 69 primer pairs that successfully amplified DNA, we chose 11 that were interspersed throughout the gene sequence. The amplified DNA was then sequenced in both forward and reverse directions for a total of 22 samples. These partial DNA sequences were examined for length, quality, and how closely they matched the reference canine *TERT* sequence.

### PCR amplification

Two sets of twenty dogs were used to derive the sequence: the first set was used to run primer pairs 1 through 40 and 66 through 75, and the second set was used to run primer pairs 41 through 65. A DNA concentration of 20 ng/μL was used for each sample for PCR, with go-Taq polymerase (Promega). PCR was performed at 42-55°C for 40 cycles. Each PCR was verified by carrying out gel electrophoresis of 8 μL of sample in a 2% agarose gel. The remaining 12 μL of successful samples were subjected to capillary sequencing (Biomedical Genomic Center, University of Minnesota).

### Sequence analysis and alignment

We compiled the resulting sequences using CodonCode Aligner (CodonCode; Dedham, Massachusetts) [14] with default parameters (Version 3.7.1.1), aligning each amplicon to the published reference sequence. The consensus sequences of each dog were aligned using clustalx (Version 2.0.11) [15]. We considered three or more identical variants from the original reference sequence to be polymorphisms. At this point each group of 20 dogs was handled separately. Gaps and ambiguous alignment segments were trimmed in Jalview^4^ (Version 11.0) [16] prior to input into three complementary programs for phylogenetic tree determination: 1. MrBayes (Version 3.2) [17], a Bayesian inference algorithm that utilizes Markov Chain Monte Carlo optimization (parameters: default General Time Reversible model of evolution, invariant sites, gamma distributed rates, 2 million generations) 2. Simple DNA parsimony as implemented in dnapars using consense (Majority Rule consensus) from the Phylip package (Version 3.69) [18]. Neighbor Joining algorithm implemented in clustalx. We visualized trees output by the tree-building programs using FigTree [19].

## Results

The DNA samples from individual dogs were prepared and PCR carried out as described in Supplementary Materials. PCR reactions were originally carried out for 74 primer pairs, and it was found that 69 of these worked on the sample dog (Additional file 1: Figure S1). It was verified that 1,245 of the 1,380 PCR samples yielded a clearly defined band of the expected size (Additional file 1: Table S1, Additional file 1: Figure S1).

**Figure 1:**
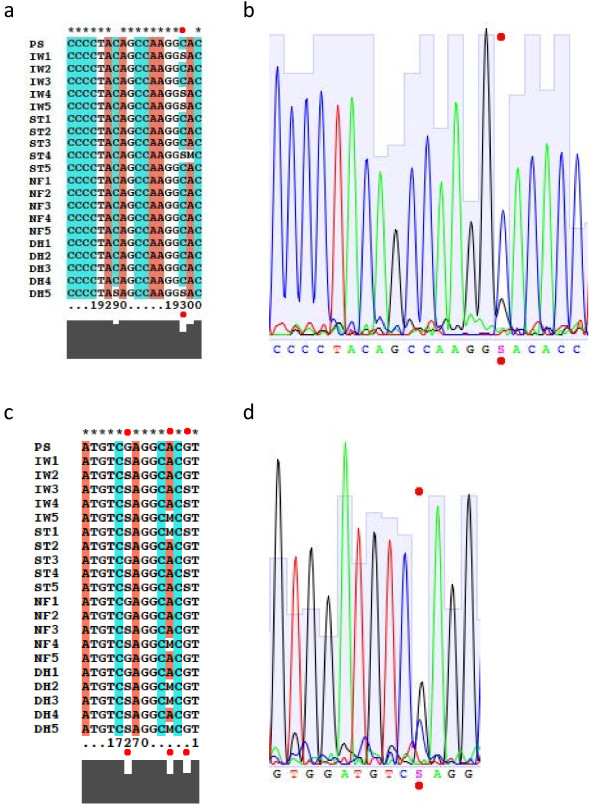
**Representative polymorphisms in the canine *TERT *gene. (a)** – Exon 17 of the *TERT* gene, displaying polymorphisms at 19289, 19298 and 19299. Location 19298 displays heterozygous polymorphisms, but no variant homozygous polymorphisms. Using the IUPAC nucleotide code, in this context “S” designates a locus that is heterozygous for G and C and “M” designates a locus that is heterozygous for A and C. **(b)** – Chromatogram of region displayed in part **(a)**, corresponding to “Irish Wolfhound 5 (IW5).” The dual peak contributing to the “S” heterozygous polymorphism is marked. **(c)** – An intronic region of the *TERT* gene, displaying polymorphisms at 17268, 17273, and 17275. These locations display heterozygous polymorphisms, but no variant homozygous polymorphisms; location 17268 is shown in the chromatogram in part **(d)**. **(d)** – Chromatogram of region displayed in part **(c)**, corresponding to “Dachshund 2 (DH2).” The dual peak contributing to the “S” heterozygous polymorphism is marked.

Clustalx was used to analyze the consensus sequence of all twenty dogs and compared them with the published sequence. As hypothesized, we identified polymorphisms within both exons and introns (Figure 1a and b). Exonic sequences within the *TERT* gene were more conserved compared to the intronic sequences. Strikingly, for many observed SNPs we found two groups: one predominantly homozyougous for the reference sequence and the other heterozygous. However, there were specific loci where the variant homozygous allele was never seen when a heterozygous allele was present. For example, at bp 19298 of the *TERT* gene (Figure 1a), located within exon 17, dogs of all breeds surveyed here were either homozygous C or heterozygous C/G (S), but none were homozygous G. It is unlikely this was the result of read contamination, as the sequence trace chromatograms for this section were clean and the peaks were clear (Figure 1b). A further example of this variation pattern can be seen at bp 17273 of the *TERT* gene (Figure 1c and d), located within an intronic region: dogs of all breeds surveyed here were either homozygous A or heterozygous A/C (M), but none were homozygous C. This variation pattern was observed throughout the gene in at least 115 instances in both sets of 20 samples, with 14 of these instances occurring in exonic regions, and 7 of those exonic variants causing a coding change. The locations of the exonic changes and their effect on codons within the gene are displayed in Table 1. A complete analysis of our sequence and the SNPs obtained is shown in Additional file 1: Figure S2 and S3.

**Table 1:** Heterozygous polymorphism in exonic sequence of TERT gene in four different dog breeds

**Exon**	**Nucleotide**	**BP change**	**Codon change**	**Individuals**
3	4395	A to M (A or C)	Thr to Pro¶	DS 1; NF 4; IW 2 & 3
3	4428	T to Y (C or T)	Cys to Arg¶	DS 1 & 3; NF 1, 3, & 5; IW 4
3	4478	T to W (A or T)	Phe to Tyr¶	DS 1; NF 3, 4 & 5
3	4517	A to M (A or C)	Ser to Ser	DS 1; NF 3; ST 1
3	4526	A to R (A or G)	Gly to Gly	DS 2; NF 1 & 2
3	4547	C to Y (C or T)	Tyr to Tyr	DS 1, 2 & 4; NF 1, 2, 3, 4, & 5; ST 3 & 5; IW 1
3	4548	A to M (A or C)	Arg to Arg	DS 4; IW 5; ST 1, 4, & 5
4	5454	A to M (A or C)	Gln to Pro¶	DS 1; NF 1 & 5; ST 1
4	5467	T to Y (C or T)	Pro to Pro	DS 1, 3, 4, & 5; NF 4 & 5; ST 2; IW 1
4	5511	A to M (A or C)	Gln to Pro¶	DS 1, 3, 4, & 5; NF 3 & 5; ST 2; IW 1
6	7125	T to Y (C or T)	Leu to Pro¶	DS 1 & 2; IW 2 & 4
12	14458	G to S (G or C)	Pro to Pro	DS 2 & 3; NF 5
17	18322	C to S (G or C)	His to Asp¶	DS 5; ST 4; IW 1, 4, &5
17	18329	G to R (A or G)	Stop to Stop	DS 5; ST 4; IW 2

¶ = Potentially deleterious mutation.

DS = Dachsund.

NF = Newfoundland.

ST = Shi Tzu.

IW = Irish Wolfhound.

**Figure 2:**
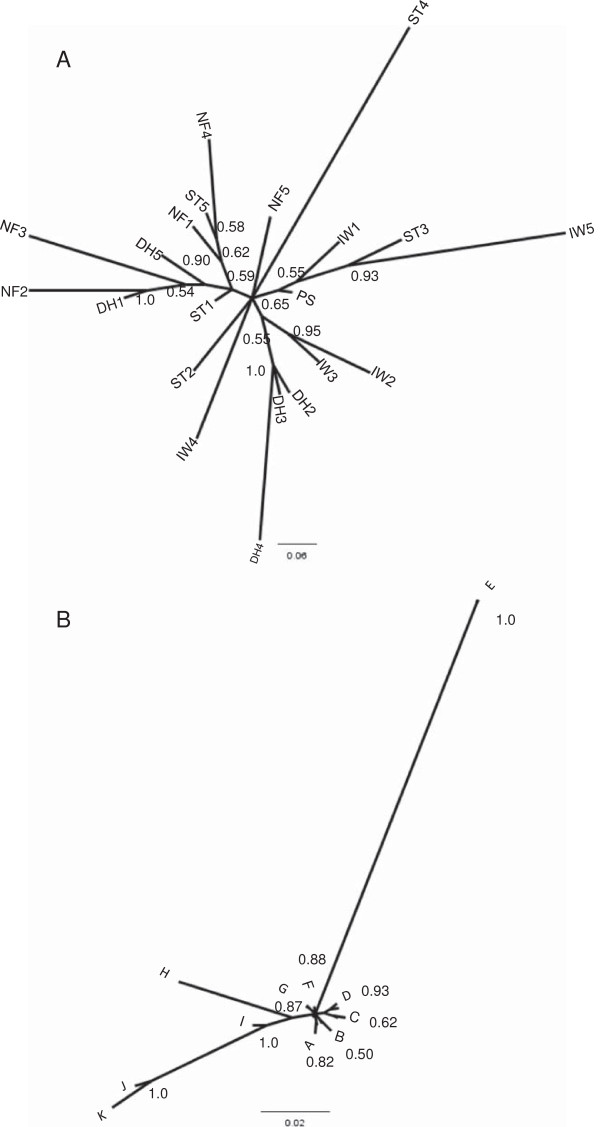
**Phylogenetic trees.** Unrooted phylogenetic trees showing relationships among **(A)** the initial set of 20 dogs sequenced from amplicons 1–40, 66–75 as displayed in Figure 1 and **(B)** a separate set of 20 dogs sequenced using amplicons 41–65. Trees were constructed using MrBayes [16] on multiple sequence alignments that were aligned with clustalx [14] and purged of gaps and ambiguous columns as outlined in Materials and Methods. Posterior probabilities for nodes with greater than 50% likelihood are indicated with branch labels. Terminal leaves are labeled according to dog breed with numbers arbitrarily assigned to individuals within a breed. Specifically, DH1-5: Dachshunds, IW1-5: Irish Wolfhounds, NF1-5: Newfoundlands, and ST1-5: Shih Tzus. There is no overlap between individuals labeled in Figures **A** and **B**. In Figure **B**, A = IW4, IW3, IW2, ST1; B = IW1, ST3; C = PS, ST4; D = NF5, DH4, DH5; E = DH2, DH3; F = ST5, NF1; G = NF4, DH1; H = NF3; I = NF2; J = ST2; K = IW5.

In analyzing the phylogenetic tree obtained from the two separate sets of dogs at the TERT locus, it was found there was no instance that placed all individuals of the same breed in one branch. Figures 2a and b illustrate this: even when as many as three members of the same breed were grouped together there was at least one other dog from a different breed in that branch. This was not an artifact of the phylogenetic inference method used, as nearly all clades within the phylogeny having high confidence (i.e., with posterior probabilities > 50%) were preserved by diverse phylogenetic methods (MrBayes, parsimony and Neighbor-joining) (data not shown).

## Discussion

We found that polymorphisms exist within both exons and introns of the *TERT* gene. A salient and unexpected observation was that in some locations, individuals were either homozygous to the reference sequence, or had polymorphisms that were heterozygous SNPs. The variant homozygote SNPs at these loci were never observed. This is likely due to the fact that dog breeds as populations are not subject to Hardy-Weinberg Equilibrium: matings are not random, and traits are deliberately selected for or against by human breeders. However, this phenomenon may also be explained by a deleterious effect of the alternative homozygous variant: it could be lethal for a conceptus. This is a definite possibility, given that inbred populations tend to have a higher concentration of deleterious recessive traits than less inbred populations [20]. Alternatively, the small sample size analyzed could be the reason for the lack of variants. In 7 of the 14 exonic occurrences, the variant caused a coding change and thus could prove deleterious. However, it is unknown if the alleles have dominant negative effects.

We expected to observe breed-specific SNPs shared amongst the majority of individuals in each breed. We also hypothesized the specific SNPs shared in each breed would be distinct from other breeds, possibly in correlation to breed size and lifespan. However, it was found that SNPs occurred in individuals without breed specificity, and there was no correlation between dog size and the observed polymorphisms; most strikingly this was seen in the close relation of the Shi Tzu and Irish Wolfhound in Figure 2b (SH2 and IW5, respectively). This lack of distinct breed association in addition to the geographic and genetic isolation of the breeds’ places of origin implies that polymorphic diversification of *TERT* preceded derivation of the four breeds studied. Despite the founder effects, population bottlenecks, and careful human selection for or against traits involved in breed creation, the *TERT* gene has remained diverse within and among dog breeds. The gene’s mutation rate and the prevalence of mutations do not seem to have been drastically altered by breed creation.

Access to breeds was limited in this study. Thus, future studies with expanded the sample sizes (individuals and breeds) and where the *TERT* genes in wolves and indigenous dogs from South East Asia were sequenced would further enhance our understanding of how and if the *TERT* gene has changed with the derivation of modern dog breeds. This also could help answer whether *TERT* is resistant to high levels of inbreeding, clarifying the actual distribution of the homozygous variants and their potential effects on viability.

## Conclusions

Our results suggest that the *TERT* gene is not wholly responsible for differences in lifespan and cancer susceptibility seen in dog breeds. Furthermore, the diversity and surprisingly higher number of SNPs in *TERT* makes us infer that mutations in this sequence are well tolerated. However, given the heterozygous SNPs that were found, we believe that the gene is not immune to the depressive effects of inbreeding. Furthermore, previous work has identified quantitative trait loci in canine chromosome 7 (CFA7) and 15 (CFA15) that were associated with size and longevity [21]. The IGF1 gene in CFA15 was proposed as one candidate having a major influence on these traits. However, the density of SNPs used in this study would not conclusively eliminate *TERT* as an additional, related or independent modulator.

Mutations in the human *TERT* gene have been shown to be important in dyskeratosis congenita, aplastic anemia, and idiopathic pulmonary fibrosis, among other disorders [22]. In addition, heterozygous mutations in the *TERT* gene have been shown to impair telomerase activity through haploinsufficiency [22]. Despite the fact that many of these disorders are heritable, it is not clear as to what extent mutations of *TERT* contribute to these diseases. Given our findings, we propose that these diseases do not arise solely due to polymorphic variants of the *TERT* gene, especially since the canine TERT protein is the closest homologue to the human protein known thus far [23].

## Availability of supporting data

The data set(s) supporting the results of this article are included within the article (and its additional files).

## Competing interests

The authors declare that they have no competing interests.

## Authors’ contributions

CAM, JFM and AB designed the experiments. CAM performed the experiments. KATS performed the bioinformatic analyses. CAM, JFM, KATS and AB analyzed the data and prepared the manuscript. All authors read and approved the final manuscript.

## Supplementary Material

Additional file 1: (Figure S1, Figure S2 and Table S1) Figure S1 Schematic representation of the PCR design and outcome. Arrows outlined in black represent amplicons that were run with a different set of DNA from the others. Green arrows represent amplicons that worked for all 20 dogs. Yellow arrows represent amplicons that failed for one or more dogs. Red arrows represent amplicons that failed for all 20 dogs. Black arrows represent amplicons that were unable to be run because no primer was available for that region of DNA. Figure S2 Nucleic acid sequence alignments used to generate phylogenetic trees in the standard interleaved NEXUS format (PMID:11975335). Standard IUPAC nucleic acid codes have been used to represent ambiguous bases. Identifiers for each dog correspond to those presented in the phylogenetic trees in Figure 2A and B respectively. As with that figure, these are arbitrary identifiers indicative of the breed (i.e., IW1-5: Irish Wolfhound; ST1-5: Shih tzu; DH1-5: Daschsund ; NF1-5: Newfoundland), and the set of dogs in A do are distinct from those in B. (A) Alignment for the first set of 20 dogs corresponding to the phylogenetic tree in Figure 2A. (B) Alignment for the second set of 20 dogs corresponding to the phylogenetic tree in Figure 2B. Table S1 Primer Pairs and PCR Conditions. The table displays each individual amplicon, and the primer pairs used to sequence it. “Yes” means the DNA successfully amplified in every dog tested; “no” means no DNA amplified in any dog tested. Additionally, the table indicates the fraction of 20 dogs for which the PCR and sequencing were successful. Click here for file
