# Correction to: Polymorphisms within the Telomerase Reverse Transcriptase gene (TERT) in four breeds of dogs selected for difference in lifespan and cancer susceptibility

**DOI:** 10.1186/s12917-021-03002-9

**Published:** 2021-09-06

**Authors:** Camille A. McAloney, Kevin A. T. Silverstein, Jaime F. Modiano, Anindya Bagchi

**Affiliations:** 1 Augsburg College, Minneapolis, MN 55454 USA; 2 College of Veterinary Medicine, University of Minnesota, St. Paul, MN 55108 USA; 3 Masonic Cancer Center, University of Minnesota, Minneapolis, MN 55455 USA; 4 Department of Veterinary Clinical Sciences, College of Veterinary Medicine, University of Minnesota, St. Paul, MN 55108 USA; 5 Department of Genetics, Cell Biology and Development, School of Medicine, 420 Delaware St. SE, MMC 806, Minneapolis, MN 55455 USA

**Correction to: BMC Vet Res 10, 20 (2014)**

**https://doi.org/10.1186/1746-6148-10-20**

The original article [1] contains an acknowledgement to Mary “Andy” Scott for graphics assistance.

The authors would like to note that this person’s name has since legally changed to Andrew Ezekiel Scott, and that the acknowledgement to Mr. Scott should now reflect their new name.
