# Supplementary material for: Polymorphisms within the Telomerase Reverse Transcriptase gene (TERT) in four breeds of dogs selected for difference in lifespan and cancer susceptibility
Source: BMC Vet Res. 2014 Jan 14;10:20. doi: 10.1186/1746-6148-10-20 (PMC3904191; doi:10.1186/1746-6148-10-20)
Supplement: Additional file 1: (Figure S1, Figure S2 and Table S1) — Figure S1 Schematic representation of the PCR design and outcome. Arrows outlined in black represent amplicons that were run with a different set of DNA from the others. Green arrows represent amplicons that worked for all 20 dogs. Yellow arrows represent amplicons that failed for one or more dogs. Red arrows represent amplicons that failed for all 20 dogs. Black arrows represent amplicons that were unable to be run because no primer was available for that region of DNA. Figure S2 Nucleic acid sequence alignments used to generate phylogenetic trees in the standard interleaved NEXUS format (PMID:11975335). Standard IUPAC nucleic acid codes have been used to represent ambiguous bases. Identifiers for each dog correspond to those presented in the phylogenetic trees in Figure 2A and B respectively. As with that figure, these are arbitrary identifiers indicative of the breed (i.e., IW1-5: Irish Wolfhound; ST1-5: Shih tzu; DH1-5: Daschsund ; NF1-5: Newfoundland), and the set of dogs in A do are distinct from those in B. (A) Alignment for the first set of 20 dogs corresponding to the phylogenetic tree in Figure 2A. (B) Alignment for the second set of 20 dogs corresponding to the phylogenetic tree in Figure 2B. Table S1 Primer Pairs and PCR Conditions. The table displays each individual amplicon, and the primer pairs used to sequence it. “Yes” means the DNA successfully amplified in every dog tested; “no” means no DNA amplified in any dog tested. Additionally, the table indicates the fraction of 20 dogs for which the PCR and sequencing were successful. [file 1746-6148-10-20-S1.pdf]

Table S1: PCR primers and conditions for PCR

Amplicon	Forward	Tm(°C)	Reverse	Tm(°C)	PCR Successful	Sequencing Successful
1	TGCACATTCTCCACCCTGCTCC	56-63	GGGTCCTTGGGTTTGTCCCTGTC	60-63	Yes	0 of 20
4	ACCAAACGCTTCCTCTACTGCTCG	54-63	GCGCAGAGCCCAGAAAGATG	60	Yes	5 of 20
5	ACCAAACGCTTCCTCTACTGCTCG	54-63	TGTGCTGTCGGAGGAGCTGTACC	60-64	Yes	13 of 20
6	TGGTACAGCTCCTCCGACAGCAC	60-64	CTGTGAGCCTGGTCCTTCCTGTAG	58-62	Yes	15 of 20
7	CTCCCTGGGAAAGCACGCTAAGCTC	60-65	AAGGATGATGAACAAGGTGCAGGTG	48-61	Yes	19 of 20
8	CCTCCCTCCCAACCATCCATCTC	60-62	TAAACATCCCACAGAACGCCCATC	50-61	Yes	16 of 20
9	CTTCCAGGCACATCTGGTCAGC	59-61	TGGCTCTAACGGACACCTCTGC	59-62	Yes	19 of 20
10	GTCCCTCCCACCTTGTTCATCTG	56-61	AGCATGGCACTTACAGTTTGTCG	47-60	Yes	12 of 20
11	AGCCAGGGTGACCTTCCAGTAAC	56-62	AGTGTGCAATGACTCGGAGAGG	54-60	Yes	15 of 20
12	TGCCCTGTACGACAACGTGGGTG	60-65	TGAGGGCCACGCATACAGAG	60	Yes	19 of 20
13	TCCTACGACCATAGCCTTGCCCAAC	56-64	TCAGATGCTCCAGGTTGGCAG	57-61	Yes	17 of 20
14	CACACATGGCTCCTCATAGGCTG	56-61	GGTCTGGTGTAGAGGACGGGAAG	60-62	Yes	15 of 20
15	ATGATAGACGCATCCTCTCCGGGTG	56-63	GACAGGCTCTCCCTATGGTCAC	59-60	Yes	18 of 20
16	AAATACAGAGGAACACAGGGAGGAC	48-60	CCTGCGTATGTTGGGCTTCG	60	Yes	20 of 20
17	ACGCAGCCCTGTCATCTCCATCC	60-65	TGCCCATCAACCAGCACAGG	60-61	Yes	18 of 20
18	AGGTTCCTGTGCTGGTTGATGG	54-61	ACTTCCAAGAGCCCAAACCTCAG	52-61	Yes	19 of 20
19	TAGGAATCAGGTGTGTTAGTCGGTG	48-60	AGCCTGCACCATCTGTCACC	60-61	Yes	2 of 20
20	TATACCGAATGGAAACCACCACCTG	48-60	TGAGTATGCTCCCTGGGCTG	60	Yes	20 of 20
21	TGCACTGCCCTTCCCTACTGAG	59-62	GGCCAGGCACCCAGAAACCTAAG	60-63	Yes	16 of 20
22	GCGTCTTAGGTTTCTGGGTGCCTGG	60-65	TGTCTCTGTGGAATGTTCTGGCTC	50-60	Yes	17 of 20
23	TGACCTCCAGACTCCGCTTCCTC	60-63	CCTGGTCTTTCTGTCCTTGGCTC	56-61	Yes	20 of 20
24	ACCCACAGGTTCTCATGGCATAG	52-60	GGAGGAAACACACAGGACGCTAAC	54-61	Yes	20 of 20
26	CACTTACGGAAGCAGAGCTGTGGAC	56-63	TTCAGTTGTGAGGTGAGATGCTGG	50-61	Yes	0 of 20
27	CGCACCTTTGTGCTACGCATAC	54-60	TGAATCAGACGCTGGAGGACC	57-60	Yes	12 of 20
28	ATTCACGCGAGTTTGGTAGTGGCAG	52-63	CATTGGCAATCACCTCTACCAGC	52-60	Yes	19 of 20
29	AAACCAGCAGTACCTGGGAGC	57-61	GCACACACACCAGTGCCACAGAC	60-64	Yes	20 of 20
30	CATCCCACGATGAGGGTGTCTG	59-61	TCACAAACCACACTCGGGAGC	57-61	Yes	16 of 20

Table S1: PCR primers and conditions for PCR

31	GGAGAAGAGAGTAGAGCAGCCGTAG	56-61	AAGCCACATCTCAGGCCCAC	60-61	Yes	13 of 20
32	GGACACAGATGGTCGCCAACAC	59-62	AAGCGAGGGCTCTTGTGCATGG	59-64	Yes	16 of 20
33	CCATGCACAAGAGCCCTCGCTTC	60-64	TGTTCATGTGTTTGTGGTGCATCC	45-60	Yes	18 of 20
34	CCTCCTCCGGTGAGATGTCAGTC	60-62	AGCGAACCAGAGACCTGGAG	60	Yes	20 of 20
35	CGAGCTTCTTCAGGTCTTGGCCTCC	60-65	ACAACAGAAACAGCAGAGCAGC	50-60	Yes	14 of 20
38	TCAGAGCTGAGAAGTAGTTGCAGG	50-60	TGCCATAGGCCACAGGAGCTGTC	60-65	Yes	20 of 20
39	CCCTACTGCCTTCTGCTCACACTCC	60-64	GGCTTGGTGGGAAGGCAGAAATG	56-62	Yes	17 of 20
40	CCCTTGTCTTTGCCAGCAGGTGTC	58-64	GCCGAGCTTGTCCCTGTAAGTG	59-61	Yes	20 of 20
41	GCCTTCCTGTGGTTCGCTAGAG	59-61	AGCTCTGGAAGAAGGCACAGAC	54-60	18 of 20	17 of 20
42	AGGCCACATGCTTACTGGCACG	59-54	AGACGCCAGAGGACCTCCCAAAC	60-64	Yes	19 of 20
44	CCGTGGTTCAGGTCATGGCTGAG	60-63	TGTTCACACACGCAACCATCC	52-60	19 of 20	7 of 20
45	GTCTTCACACAGATAGCTTGCCTGG	52-61	TACCCGTCCTGCTCGATTCC	60	19 of 20	8 of 20
46	TCCCTCAGGGCTCTATCCTGTCCAC	60-65	CCAAAGGATCTGGACAATGGCTG	52-60	Yes	8 of 20
48	CCCTATGCGTCTCTGTGGCACTC	60-63	AATGCACCAAGCTCCCAAGTGGTC	54-64	Yes	9 of 20
50	AATTCAGTCGGCACAAGGGCTCC	56-63	TGGTTCGCTTCCTGCCACAC	60-61	Yes	7 of 20
52	ACACACAGGCTGCTGTCACG	60-61	GCGCTCATTCTGTGCTACGTGG	59-62	Yes	7 of 20
53	CGTCCAGACCCTGCATTCTCCTC	60-63	GTCTTCTGCAAGTTGGCCCTG	57-60	Yes	0 of 20
54	GCAGGGCCAACTTGCAGAAGACG	60-64	TGTGCCCTTGTCCTGAGGAG	60	19 of 20	19 of 20
55	GCTCCCACTCCTGTCTCCACAAG	60-63	CGCAGAGAGACGTGAGGACACAG	60-62	17 of 20	16 of 20
56	CCACTGCACCAGACTCTGTCCTCAC	60-64	AGCAGATTCCAGCACCCACGTCC	60-65	Yes	20 of 20
58	CCGTCTGCACTTGGACCTGTCTC	60-63	GAAGATGGGTGCAGGCGAGGTTG	60-64	17 of 20	8 of 20
59	GTCTTGCGGCTGAAGTGCTG	60	ATAGACCGTGTGGATGCCGTTC	54-61	Yes	9 of 20
60	GCCACTTGGCGCTCACATTCTG	59-62	GGGACGGACACCTCCAATGAAG	59-61	Yes	17 of 20
61	CAGGCTTACAGGTGGGTTCACG	59-61	GGGACGGACACCTCCAATGAAG	59-61	Yes	18 of 20
62	TTGGCGGTGGAGCCTTCCTAACAG	58-65	ACAGACTCACCATAGCTCCACAC	52-60	Yes	8 of 20
63	AGCCGGGCTTTGCTGGTTTCTG	59-64	TGCGTGGTTGGAGGCAACAG	60-62	Yes	11 of 20
64	GTGCATTTGTTGTGCAGTGAGCTTC	48-61	TGAGCGTCCACCACCTCCAATC	59-63	Yes	20 of 20
66	GATACCTGCCCACCGGGAAATG	59-61	TATGGGTGGAGGACCCTCTCTG	59-61	Yes	20 of 20

Table S1: PCR primers and conditions for PCR

67	CCCAAAGTGGGACACTAGCTCATGC	56-63	GACTGGAGCCAGGGTTGGTAATG	56-61	Yes	20 of 20
68	GTCTGTGCATTACCAACCCTGGCTC	56-63	AAGCACCCATGTGACTCCACAAAC	50-62	Yes	19 of 20
69	TGCATGTGTGCTCCAGCTTCC	57-62	GGGACACACAGCATAGCCTGAAC	56-62	Yes	20 of 20
70	CTGTGTGGCTGCTCGTCCATCTCTG	60-65	GGCTTCAGAAGGAAACAGGCCAG	56-62	Yes	18 of 20
72	TCCACGCCTTCCTGCTCAAGCTG	60-65	AGCTGGTATGGGTGGGAGAGAG	59-61	Yes	15 of 20
73	ACCTGGTCACTCCACACCTCACCTC	60-65	TCAGGTGTGCCTTGGCTGTAG	57-61	Yes	20 of 20
75	TGAGGCCCTGAGAGTTGAGCTGC	60-65	GACACAGACTTTGGACGCAGC	57-60	16 of 20	0 of 20

Figure S1

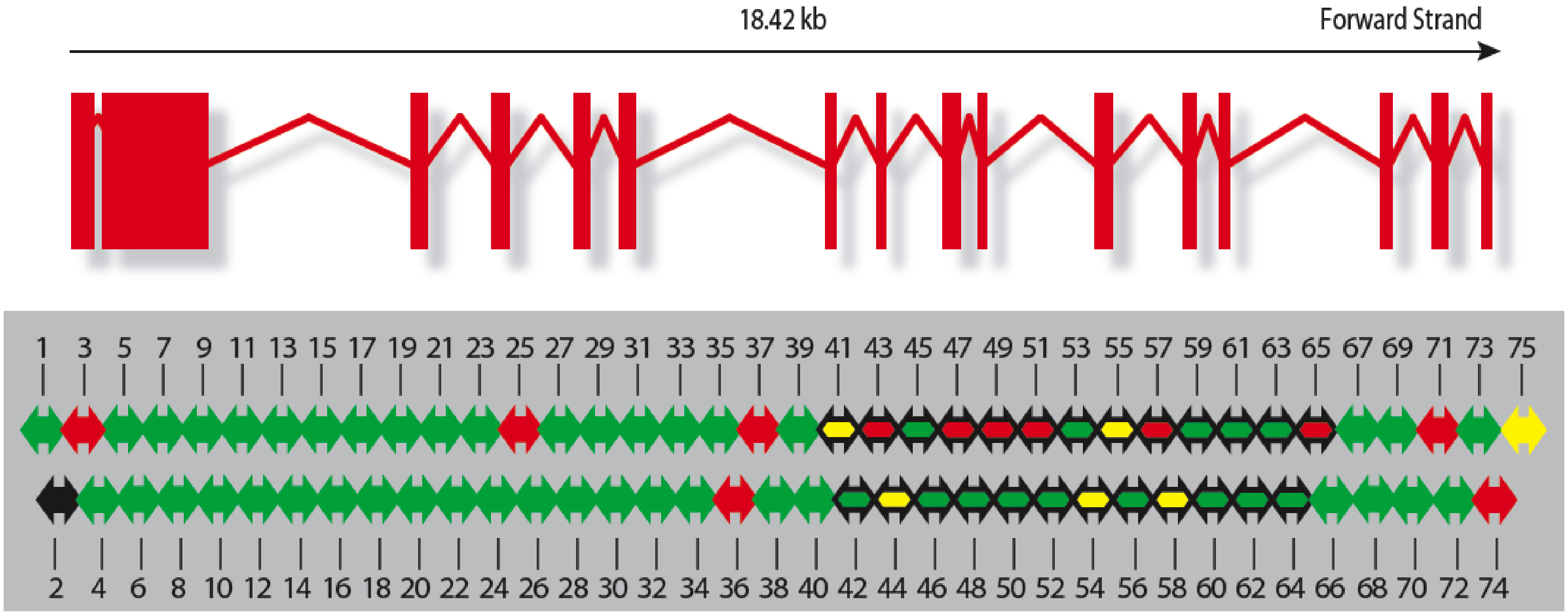

18.42 kb
Forward Strand
1 3 5 7 9 11 13 15 17 19 21 23 25 27 29 31 33 35 37 39 41 43 45 47 49 51 53 55 57 59 61 63 65 67 69 71 73 75
2 4 6 8 10 12 14 16 18 20 22 24 26 28 30 32 34 36 38 40 42 44 46 48 50 52 54 56 58 60 62 64 66 68 70 72 74

Figure S2

```
#NEXUS
begin data;
dimensions ntax=21 nchar=1754;
format datatype=dna interleave=yes gap=-;

matrix
PS             CCCCTCCCGTTTACGTGAACGTTGGAGATGCTTGTGGGTGCAGGCACGCC
IW1            CCYCTCCCKTTTHCGTGAACGTTGGAGATGCTTGTGGGTGCAGGCACGCC
IW2            CCCBTCCBGSTTACGTGAACGTTGGAGATGCTTGTGGGTGCAGGCACGCC
IW3            CCCCTCCC-TTTACGTGAACGTTGGAGATGCTTGTGGGTGCAGGCACGCC
IW4            CCCYTCCCGTTTACGTGAACGTTGGAGATGCTTGTGGGTGCAGGCACGCC
ST3            YCCHTCCCGTTTWCGTGAACGTTGGAGATGCTTGTGGGTGCAGGCACGCC
IW5            YCCCTCCCGTTTACGTGAACGTTGGAGATGCTTGTGGGTGCAGGCACGCC
ST2            CCCHTCCCGTTTACGDKRACGTTGGAGATGCTTGTGGGTGCAGGCACGCC
ST1            CCCCTCCCGTTTACGTGAACGTTGGAGATGCTTGTGGGTGCAGGCACGCC
DH1            YCCCTCCSGTTTACGTGAACGTTGGAGATGCTTGTGGGTGCAGGCACGCC
NF2            CCCCTCCSGTTTACGTGAACGTTGGAGATGCTTGTGGGTGCAGGCACGCC
NF1            YCCCTCCGGTTT-CGKGAACGTTGGAGATGCTTGTGGGTGCAGGCACGCC
NF3            BCCCTCCCDTTTACGTGAACGTTGGAGATGCTTGTGGGTGCAGGCACGCC
NF5            MCCYTCMYSTYTACGHBARYDTSAGKGATGCYTDYGGGTGCAGGCACSCC
ST4            TTCCRTCCGTTTACGTG-ACGTTGGAGATGCTTGTGGGTGCAGGCACGCC
ST5            YCCCTCCSGTTTACGTGAACGTTGGAGATGCTTGTGGGTGCAGGCACGCC
NF4            YCCYTCCVGTTTASGTGAACGTTGGAGATGCTTGTGGGTGCAGGCACGCC
DH2            CCCCTCCCGTTTACGTGAACGTTGGAGATGCTTGTGGGTGMAGGCACSCC
DH3            CCCCTCCCGTTTACGTGAACGTTGGAGATGCTTGTGGGTGCAGGCACGCC
DH4            CCCCTCCCGTTTACGTGAACGTTGGAGATGCYTGTGGGTGMAGGCACGCC
DH5            CCCCTCCCGTTTACGTGAACGTTGGAGATGYTTGWGGKTGCAGGCACGCC
```

PS	CTGGACCCAAGCAGTGAGCACGTGTGATGTGCATGGACACGCCCACAGCA
IW1	CTGGACCCAAGCAGTGAGCACGTGTGATGTGCATGGACACGCCCACAGCA
IW2	CTGGACCCAAGCAGTGAGCACGTGTGATGTGCATGGACACGCCCACAGCA
IW3	CTGGACCCAAGCAGTGAGCACGTGTGATGTGCATGGACACGCCCACAGCA
IW4	CTGGACCCAAGCAGTGAGCACGTGTGATGTGCATGGACACGCCCACAGCA
ST3	CTGGACCCAAGCAGTGAGCACGTGTGATGTGCATGGACACGCCCACAGCA
IW5	CTGGACCCAAGCAGTGAGCACGTGTGATGTGCATGGACACGCCCACAGCA
ST2	CTGGACCCAAGCAGTGAGCACGTGTGATGTGCATGGACACGCCCACAGCA
ST1	CTGGACCCAAGCAGTGAGCACGTGTGATGTGCATGGACACGCCCACAGCA
DH1	CTGGACCCAAGCAGTGAGCACGTGTGATGTGCATGGACACGCCCACAGCA
NF2	CTGGACCCAAGCAGTGAGCACGTGTGATGTGCATGGACACGCCCACAGCA
NF1	CTGGACCCAAGCAGTGAGCACSTGTGATGTGCATGGACACGCCCACAGCA
NF3	CTGGACCCAAGCAGTGAGCACGTDTGATGTGCATGGACACGCCCACAGCA
NF5	CTGGACCCAAGCAGTGAGCACGTGTGATGTGCATGGACACGCCCACAGCA
ST4	CTGGACCCAAGCAGTGAGCACGTGTKATGTGCATGGACACGCCCACAGCA
ST5	CTGGACCCAAGCAGTGAGCACGTGTGATGTGCATGGACACGCCCACAGCA
NF4	CTGGACCCAAGCAGTGAGCACGTGTGATGTGCATGGACACGCCCACAGCA
DH2	CTGGACCCAAGCAGTGAGCACGTGTGATGTGCATGGACACRCCCACAGCA
DH3	CTGGACCCAAGCAGTGAGCACGTGTGATGTGCATGGACACGCCCACAGCA
DH4	CTGGACCCAAGCAGTGAGCACGTGTGATGTGCATGGACACGCCCACAGCA
DH5	CTGGAHCCAAGCAGTGAGCACGTGTGATGTGCATGGACACGCCCACAGCA

PS	ACCGGTGATGCGTCCAGAAGCTCTCACAAGCACACCTGCACCTTGTTCAT
IW1	ACCGGTGATGCGTCCAGAAGCTCTCACRAGCACACCTGCACCTTGTTCAT
IW2	ACCGGTGATGCGTCCAGAAGCTCTCACAAGCACACCTGCACCTTGTTCAT
IW3	ACCGGTGATGCGTCCAGAAGCTCTCACAAGCACACCTGCACCTTGTTCAT
IW4	ACCGGTGATGCGTCCAGAAGCTCTCACRAGCACACCTGCACCTTGTTCAT
ST3	ACCGGTGATGCGTCCAGAAGCTCTCACRAGCACACCTGCACCTTGTTCAT
IW5	ACCGGTGATGCGTCCAGAAGCTCTCACRAGCACACCTGCACCTTGTTCAT

ST2 ACCGGTGATGCGTCCAGAAGCTCTCACAAGCACACCTGCACCTTGTTCAT

ST1 ACCGGTGATGCGTCCAGAAGCTCTCACRAGCACACCTGCACCTTGTTCAT

DH1 ACCGGTGATGCGTCCAGAAGCTCTCACAAGCACACCTGCACCTTGTTCAT

NF2 ACCGGTGATGCGTCCAGAAGCTCTCACAAGCACACCTGCACCTTGTTCAT

NF1 ACCGGTGATGCGTCCAGAAGCTCTCACGAGCACACCTGCACCTTGTTCAT

NF3 ACCGGTGATGCGTCCAGAAGCTCTCACRAGCACACCTGCACCTTGTTCAT

NF5 ACCGGTGATGCGTCCAGAAGCTCTCACAAGCACACCTGCACCTTGTTCAT

ST4 ACCGGTGATGCGTCCAGAAGCTCTCACAAGCACACCTGCACCTTGTTCAT

ST5 ACCGGTGATGCGTCCAGAAGCTCTCACGAGCACACCTGCACCTTGTTCAT

NF4 ACCGGTGATGCGTCCAGAAGCTCTCACRAGCACACCTGCACCTTGTTCAT

DH2 ACCGGTGATGCGTCCAGAAGCYCYCACRAGCACACCTGCACCTTGTTCAT

DH3 ACCGGTGATGCGTCCAGAAGCTCTCACRAGCACACCTGCACCTTGTTCAT

DH4 ACCGGTGATGCGTCCAGAAGCTCTCACRAGCACACCTGCACCTTGTTCAT

DH5 ACCGGTGATGCGTCCAGAAGCTCTCACAAGCACACCTGCACCTTGTTCAT

PS CATCCGCGCGGCGGGACTTCAGGAGGGGAGAGGTCTCCTGACAAGCCCCC

IW1 CATCCGCGCGGCGGGACTTCAGGAGGGGAGAGGTCTCCTGACAAGCCCCC

IW2 CATCCGCGCGGCGGGACTTCAGGAGGGGAGAGGTCTCCTGACAAGCCCCC

IW3 CATCCGCGCGGCGGGACTTCAGGAGGGGAGAGGTCTCCTGACAAGCCCCC

IW4 CATCCGCGCGGCGGGACTTCAGGAGGGGAGAGGTCTCCTGACAAGCCCCC

ST3 CATCCGCGCGGCGGGACTTCAGGAGGGGAGAGGTCTCCTGACAAGCCCCC

IW5 CATCCGCGCGGCGGGACTTCAGGAGGGGAGAGGTCTCCTGACAAGCCCCC

ST2 CATCCGCGCGGCGGGACTTCAGGAGGGGAGAGGTCTCCTGACAAGCCCCC

ST1 CATCCGCGCGGCGGGACTTCAGGAGGGGAGAGGTCTCCTGACAAGCCCCC

DH1 CATCCGCGCGGCGGGACTTCAGGAGGGGAGAGGTCTCCTGACAAGCCCCC

NF2 CATCCCCCCGGCGGGACTTCAGGAGGGGAGAGGTCTCCTGACAAGCCCCC

NF1 CATCCGCGCGGCGGGACTTCAGGAGGGGAGAGGTCTCCTGACAAGCCCCC

NF3 CATCCGCGCGGCGGGACTTCAGGAGGGGAGAGGTCTCCTGACAAGCCCCC

NF5 CATCCGCGCGGCGGGMATTCAGGAGGGGAGAGGTCTCCTGACAAGCCCCC

ST4 CATCCGCGCGGCGGGACTTCAGGAGGGGAGAGGTCTCCTGACAAGCCCCC

ST5 CATCCDCGCGGCGGGACTTCAGGAGGGGAGAGGTCTCCTGACAAGCCCCC

NF4 CATCCGCGCGGCGGGACTTCAGGAGGGGAGAGGTCTCCTGACAAGCCCCC

DH2 CATCCGCRCGGCGGGAATTCAGGAGGGGAGAGGTCTCCYGACAAGCCCCC

DH3 CATCCGCGCGGMRGGASTTMAGGAGGGGAGWGGTCTCCYGACAAGCCCCC

DH4 CATCCGCGCKSCGGGASTTYAGGAGGGGAGTGGTCTCCYGAYAAGCCCCC

DH5 CATCCGCGCGGCGGGACTTCAGGAGGGGAGAGGTCTCCTGACAAGCCCCC

PS CCCCCCCCGCCCCCCCCCCTTGCCCCCAAGCGCTTAAAAGGAGAGTTCCA

IW1 CCCCCCCCGCCCCCCCCCCTTGCCCCCAAGCGCTTAAAAGGAGAGGTCCA

IW2 CCCCCCCCGCCCCCCCCCCTTGCCCCCAAGCGCTTAAAAGGAGAGWRWCM

IW3 CCCCCCCCGCCCCCCCCCCTTGCCCCCAAGCGCTTAAAAGGAGAGTTCCA

IW4 CCCCCCCCGCCCCCCCCCCTTGCCCCCAAGCGCTTAAAAGGAGAGTTCCA

ST3 CCCCCCCCGCCCCCCCCCCTTGCCCCCAAGCGSTTAAAAGGAGARKTCCA

IW5 CCCYAMCCGCCCCCCCCCCTTGCCCCCAAGCGCTTAAAAGGAGAGGTTCM

ST2 CCCCCCCCGCCCCCCCCCCTTGCCCCCAAGCGCTTAAAAGGAGAGTTCCA

ST1 CCCCCCCCGCCCCCCCCCCTTKCCCCCAAGCGCTTAAAAGGAGARTTCCA

DH1 CCCCCCCCGCCCCCCCCCCTTGCCCCCAAGCGCTTAAAAGGAGAGTBCCA

NF2 CCCCCCCCGCCCCCCCCCCTTGCCCCCAAGCGCTTAAAAGGAGAGGTCCM

NF1 CCCCCCCCGCCCCCCCCCCTTGCCCCCAAGCGCTTAAAAGGAGARTTCCA

NF3 CCCCCCCCGCCCCCCCCCCTTGCCCCCAADCGCTTAAAAGGAGARTTCCA

NF5 CCCCCCCCGCCCCCCCCCCTTGCCCCCAAGCGCTTAAAAGGAGAGKTCCA

ST4 CCCCCCCCGCCCCCCCCCCTTGCCCCCAAGCGCTTAAAAGGAGAGYTCCA

ST5 CCCCCCCCGCCCCCCCCCYTTGCCCCMAAGCGCTTAMARGGAGAGTTCCA

NF4 CCCCCCCCGCCCCCCCCCCTTGCCCCCAAGCGCTTAAAAGGAGAGTTCCA

DH2 CCCGCCCCGCCCCCCCCCCTTKCCCCCMARCGGYTAAAAGGGGAATTCCA

DH3 CCCGCCCCGCCCCCCCCCYTTKCCCCCMARCGGYTAAAAGGRGAATTCMA

DH4 CCCGCCCCKYCCCCCCCCCTTKCCCCCMARCGSTTAAAAGGRGAATTCCA

DH5 CCCCCCCCGCCCCCCCCCCTTGCCCCCAAGCGCTTAAAAGGAGAGTTCCA

PS	CTGCGGGACATGTGGCTGCCTCTCTTGCCTGTGGGGTTCAGCCTGGAGAA
IW1	CTGCGGGACATGTGGCTGCCTCTCTTGCCTGTGGGGTTCAGCCTGGAGAA
IW2	CTSCSGGA-ATGTGGCTGYCTCTSTTGCCTGKGGGGTTCASCCTGRAKAA
IW3	CTGCGGGACATGTGGCTGCCTCTCTTGCCYGTGGGGTTCAGCCTGGAGAA
IW4	CTGCGGGACATGTGGCTGCCTCTCTTGCCTGTGGGGTTCAGCCTGGAGAA
ST3	CTGCGGGACATGTGGCTGCCTCTCTTGCCTGTGGGGTTCAGCCTGGAGAA
IW5	CTGCGKGA-RTGTGGCTGCCTCTSTTGCCTGKGGGGTTCASCCTGGAKAA
ST2	CTGCGGGACATGTGGCTGCCTCTCTTGCCTGTGGGGTTCAGCCTGGAGAA
ST1	CTGCGGGACATGTGGCTGCCTCTCTTGCCTGTGGGGTTCAGCCTGGAGAA
DH1	CTGCGGGACATGTGGCTGCCTCTCTTGCCTGTGGGGTTCAGCCTGGAGAA
NF2	CTGCGGGACATBTGGCTGCCTCTCTTGCCHGTGGGGTTCAGCCTGGAGAA
NF1	CTGCCGGACMTGTGGCTGHCTCYCTTGCCHGTGGGGTTCAGCCTGGAGAA
NF3	CTGCGGGACATGTGGCTGCCTCTCTTGCCTGTGGGGTTCAGCCTGGAGAA
NF5	CTGCGGGACATGTGGCTGCCTCTCTTGCCTGTGGGGTTCAGCCTGGAGAA
ST4	CTGCGGGACATGTGGCTGCCTCTCTTGCCTGTGGGGTTCAGCCTGGAGAA
ST5	CTGCGGGACATGTGGCTGCCTCTCTTGCCTGTGGGGTTCAGCCTGGAGAA
NF4	CTGCSGGACATGTGGCTGCCTCTCTTGCCTGTGGGGTTCAGCCTGGAGAA
DH2	CTGCGGGACATGTGGCTGCCTCTCTTGCCTGTGGGGTTCAGCCTGGAGAA
DH3	CTGCGGGACATGTGGCTGCCTCTCTTGCCTGTGGGGTTCAGCCTGGAGAA
DH4	CTGCGGGACATGTGGCTGCCTCTCTTGCCTGTGGGGTTCAGCCTGGAGAA
DH5	CTGCGGGACATGTGGCTGCCTCTCTTGCCTGTGGGGTTCAGCCTGGAGAA

PS	CCCCAGGCATTCTGGACGGGATCCAGTGATGCTGCTTAAGGACTGCACGT
IW1	CCCCAGGCATTCTGGACGGGATCCAGTGATGCTGCTTAAGGACTGCACGT
IW2	CCCCAGGCATTCKGGACGGRATCCAGTGATGCTGCTTAASGACTGCMCGT
IW3	CCCCAGGCATTCTGGACGGGATCCAGTGATGCTGCTTAAGGACTGCACGT
IW4	CCCCAGGCATTCTGGACGGGATCCAGTGATGCTGCTTAAGGACTGCACGT
ST3	CCCCAGGCATTCTGGACGGGATCCAGTGATGCTGCTTAAGGACTGCACGT

IW5	CCCCAGGCATTCKGGACRGGATCCAGTGATGCTGCTTAAKGACTGCMCGT
ST2	CCCCAGGCATTCTGGACGGGATCCAGTGATGCTGCTTAAGGACTGCACGT
ST1	CCCCAGGCATTCTGGACGGGATCCAGTGATGCTGCTTAAGGACTGCACGT
DH1	CCCCAGGCATTCTGGACGGGATCCAGTGATGCTGCTTAAGGACTGCACST
NF2	CCCCMGGCATTCTGGACGGGATCCAGTGATGCTGCTTAAGGACTGCACGT
NF1	CCCCAGGCATTCTGGACDGGATCCAGTGATGCTGCYTAAGGACTGCACGT
NF3	CCCCAGGCATTCTGGACGGGATCCAGTGATGCTGCTTAAGGACTGCACGT
NF5	CCCCAGGCATTCTGGACGGGATCCAGTGATGCTGCTTAAGGACTGCACGT
ST4	CCCCAGGCATTCTGGACGGGATCCAGTGATGCTGCTTAAGGACTGCACGT
ST5	CCCCAGGCATTCTGGACGGGATCCAGTGATGCTGCTTAAGGACTGCACGT
NF4	CCCCAGGCATTCTGGACGGGATCCAGTGATGCTGCTTAAGGACTGCACGT
DH2	CCCCAGGCATTCTGGACGGGATCCAGTGATGCTGCTTAAGGACTGCACGT
DH3	CCCCAGGCATTCTGGACGGGATCCAGTGATGCTGCTTAAGGACTGCACGT
DH4	CCCCAGGCATTCTGGACGGGATCCAGTGATGCTGCTTAWKGACTGCMCGT
DH5	CCCCAGGCATTCTGGACGGGATCCAGTGATGCTGCTTAAGGACTGCACGT

PS	GACCATAGGGAGAGCCTGTCTCTGCGTCTCCATCCTACTCATAGATAAGA
IW1	GACCATAGGGAGAGCCTGTCYCTGCSTCYCCATCCTACTCATAGATAARA
IW2	GACCATAGGGAGAGCCTGTCTCTGCSTCYCCATCCTACTCATAGATAARA
IW3	GACCATAGGGAGAGCCTGTCTCTGCSTCYCCATCCTACTCATAGATAARA
IW4	GACCATAGGGAGAGCCTGTCYCTGCSTCYCCATCCTACTCATAGATAARA
ST3	GACCATAGGGAGAGCCTGTCTCTGCSTCTCCATCCTACTCATAGATAARA
IW5	GACCATASGGAGAGCCTGTCTCTGCRTCYCCATCCTACTCATAGATAARA
ST2	GACCATAGGGAGAGCCTGTCTCTGCGTCTCCATCCTACTCATAGATAARA
ST1	GACCATAGGGAGAGCCTGTHTCTGCSTCTCCATCCTACTCATAGATAARA
DH1	GACCATASGGAGAGCCTGTCYCTSCRTCTCCATCCYACTCATAGATAAAA
NF2	GACCATASGGAGAGCCTGTCBBTGCSTCYCCATCCTACTCATAGATAARA
NF1	GACCATAGGGAGAGCCYGTCCVBGCSTCTCCATCCYACTCATAGATAAAA
NF3	GACCATAGGGAGAGCCTGTCTVTGCSTCTCCATCCTACTCATAGATAARA

NF5	GACCATAGGGAGAGCCTGTCBVTGCSTCTCCATCCYACTCATAGATAARA
ST4	GACCATAGGGAGAGCCTGTCTCWGCGTCTCCATCCTACTCATAGATAARA
ST5	GACCATAGGGAGAGCCTGTCBVTGCSTCTCCATCCTACTCATAGATAARA
NF4	GACCATAGGGAGAGCCTGTCBCTGCSTCYCCATCCTACTCATAGATAARA
DH2	GACCATAGGGAGAGCCTGTCTCTGCRTCTCCATCCTACTCATAGATAARA
DH3	GACCATAGGGAGAGCCTGTHTBHGCGTCYCCATCCTACTCATAGATAARA
DH4	GACCATASGGAGAGYCTGTCTCTGCMTSYCCATCCYACTCATAGATAARA
DH5	GACCATAGGGAGAGCCTGTCTCTGCSTCTCCATCCTACTCATAGATAARA

PS	ACTGATGACGCCCCTGTCATCTCCTCCGAAGTAAAAAACAAAATGGGAAA
IW1	ACTGAKGACGCCCCTGTCMTCTCCTCCGAADTAAAAAACAAVATGGGAAA
IW2	ACTGAKGACSCCCCTGTCATCTCCTCCGHAGTAAAAAACAAAATGGGAAA
IW3	ACTGATGACKCCCCTGTCATCTCCTCCGAAGTAAAAAACAADATBGGAAA
IW4	ACTGAKGACKCCCCTGTCATCTCCTCCGAVGTAAAAAACAAAATGGGAAA
ST3	ACTGAYGACKCCCCTGTCATCTCCTCCGAAGTAAAAAACAAAATGGGAAA
IW5	ACTGAKGACKCCCCTGTCATCTCCTCCGAAGTAAAAAACAAAATBGGAAA
ST2	ACTGAKGACSCCCCTGTCATCTCCTCYGAAGTAAAAAACAAAATGGGAAA
ST1	ACTGAKGACKCCCMTGTCATCTCCYCCGAAGTAAAAAACAAAATGGGAAA
DH1	ACTGAYGACSCCCCTGTCATCTCCTCCGAAGTAAAAAACAAAATGGGAAA
NF2	ACTGAKGACKCCCCTGTCATCTCCTCCGAAGTAAAAAACAAAATGGGAAA
NF1	ACTGAKGACSCCCCTGTCMTCYCCTCCGAAKTAAAAAACDWAATGSSWWA
NF3	ACTGAKGACKCCCCTGTCATCTCCTCCGMAGTAAAAAACAAWWTGGGHWA
NF5	ACTGAKGACKCCCCTGTCATCTCCTYCGAAGTAAAAAACAAAATGGGAAA
ST4	ACTGACGACGCCCCTGTCATCTCCTCCGAAGTAAAAAACAAAATGGGAAA
ST5	ACTGATGACKCCCCTGTCATCTCCTCCGAAGTAAAAAACAAAATGGGAAA
NF4	ACTGWKGACKCCCMTGTCATCTCCTCCGADVTAAAAADCAAAATGGGAAA
DH2	ACTGAYGACKCCCMTGTCATCTCCTCYGAWGTAAAAAACAAAATGGGAAA
DH3	ACTGAYGACKCCCCTGTCMTCTCCTCCGAAGTVAAAAACAAAATGGGAAA
DH4	ACTSAKGACKCCCCTGTCMTCTCCTCCGAAGTAVAAAACAAAATGGGAAA

```
DH5         ACTGAYGACKCCCCTGTCATCTCCYCYGAAGTAAAAAACAAAATGGGAAA

PS          CAAATCTAATAGTCTTTTGTCAAGCCCAACATACGCAGGACGGTGCCTAA
IW1         CAAATCTAATAGTCTTTTGTCAAGCCCAACATACGCAGGACGGTGCCTAA
IW2         CAAATCTAATAGTCTTTTGTCAAGCCCAACATACGCAGGACGGTGCCTAA
IW3         CAAATCTAATAGTCTTTTGTCAAGCCCAACATACGCAGGACGGTGCCTAA
IW4         CAAATCTAATAGTCTTTTGTCAAGCCCAACATACGCAGGACGGTGCCTAA
ST3         CAAATCTAATAGTCTTTTGTCAAGCCCAACATACGCAGGACGGTGCCTAA
IW5         CARATCTAATAGBCTTTTGTCAAGCCCAACATACGCAGGACGGTGCCTAA
ST2         CAAATCTAATAGTCTTTTGTCAAGCCCAACATACGCAGGACGGTGCCTWA
ST1         CAAATCTAATAGTCTTTTGTCAAGCCCAACATACGCAGGACGGTGCCTAA
DH1         CARATCTRATAGTCBTTTGTCAAGCCCAACATACGCAGGACGCTGCCGAA
NF2         CAAADCTRATWGTCTHTTGTCAAGCCCAACATACGCAGGACGYTGCCGAA
NF1         CWRATCTRVTWGKCTHTHGTCAAGCCCVACATACCCAGGACGGTGCCTAA
NF3         CAAATCTAATWGKVTHTTGTCAAGCCCAACATACGCAGGACGCTGCCGAA
NF5         CAAATCTAATAGTCTTTTGTCAAGCCCAACATACGCAGGACGGTGCCTAA
ST4         CAAATCTAATASTCTTTTGTCAAGCCCAACATACGCAGCCCGGTGCCTAA
ST5         CAAATCTAATAGTCTTTTGTCAAGCCCAACATACGCAGGACGGTGCCTAA
NF4         CARATCTAATAGTCHTTTGTCAAGCCCAACATACGCAGGACGGTGCCTAA
DH2         CHRATCTAVTAGTCTTTTGTCAAGCCCAACATACGCAGGACGGTGCCTWH
DH3         CAAATCTAATAGTCTTTTGTCAAGCCCAACATACGCAGGACGGTGCCYAA
DH4         CARATCTAATAGTCTTTTGTCAAGCCCAACATACGCAGGACGGTGCCTHS
DH5         CAAATCTAATAGTCTTTTGTCAAGCCCAACATACGCAGGACMCMGGCCAA

PS          AGTGTTAACCCCAGGGCCTGCGGGCCGGGTGCCCGAGTGCAGAGGACTGG
IW1         AGTGSTAACCCCAGGGCCTGCGGGCCGGGTGCCCGASTGCRRAGGACTGG
IW2         AGTGTTAACCCCAGGGCCTGCGGGCCGRSWGMCMGASYGCRRAGGACTGG
IW3         AGTGTTRACCCCAGGGCCTGCGGGCCGGGTGMCCGASTGCRRAGGACTGG
IW4         AGTGTTAACCCCAGGGCCTGCGGGCCGGGTGCCCGAGTGCRGAGGACTGG
```

ST3	AGTGKTAACCCCAGGGCCTGCGGGCCGGGTGMCCGASTGCRRAGGACTGG
IW5	AGTGTTRACCCCAGGGCCTGCGGGMCGGGTGMCCGASTGCRRAGGACTGG
ST2	AGTGTTDWCYCYGGGWCCWGYSVSCCSSCHSCCBGAGTBCAAAGGGTGAG
ST1	AGTGTTAACCCCAGGGCCTGCGGGMCGGGWGMCCGASTRCRRAGGACTGG
DH1	AGTBDTVWYYYCAGGRCCYBCBKKCCGGGTGCCCGASTGCRGAGGACTGG
NF2	AGTGTTAACCCCAGGGCCTGCGGGCCGGGTGCCCGASTGCRRAGGACTGG
NF1	AGTGTTAACCCCAGGGCCTGCGGGCCGGGTGCCCGASTGCRGAGGACTGG
NF3	AGTGTTAACCCCAGGGCCTGCGGGCCGGGTGMCCSASTGCRRAGGACTGG
NF5	AGTGKTAACCCCAGGGCCTGCGGGCCGGGTGCCMGASTGCRGAGGACTGG
ST4	AGTGTTAACCCCAGGGCCTGCGGGCCGGGTGCCCGASTGCRGAGGACTGG
ST5	AGTGSTAACCCCAGGGCCTGCGGGCCSGGTGCCCGASTGCRGAGGACTGG
NF4	AGTGGTSACCCCAGGGCCTGCGGGCCGRGTGMCCGASTGSRRAGGACTGG
DH2	MGTGTTMMCCCCAGGGCCYSCGGGCCGGGTGCCCGASTGCRRAGGACTGG
DH3	AGTGSTAACCCCAGGGCCTGCGGGCCGGGTGCCCGASTGCRRAGGACTGG
DH4	YACKWCHTCCCAAGGGCCTGCGGGCCGGGTGCCCGASTGCRRAGGACTGG
DH5	AGTGKTAACCCCAGGGCCTGCGGGCCGGGTGCCCGASTGCRGAGGACTGG

PS	TAGGAAGGCCGGTCCTAGGAGATGCTGGGGCTTCCATGAACCCACAGGTT
IW1	TAGGAAGGCCGGTCCTAGGAGATGCTGGGGCTTCCATGAACCCACAGGTT
IW2	TAGGAAGGCCGGTCCTAGGAGATGCTGGGGCTTCCATGAACCCACAGGTT
IW3	TAGGAAGGCCGGTCCTAGGAGATGCTGGGGCTTCCATGAACCCACAGGTT
IW4	TAGGAAGGCCGGTCCTAGGAGATGCTGGGGCTTCCATGAACCCACAGGTT
ST3	TAGGAAGGCCGGTCCTAGGAGATGCTGGGGCTTCCATGAACCCACAGGTT
IW5	TAGGAAGGCCGGTCCTAGGAGATGCTGGGGCTTCCATGAACCCACAGGTT
ST2	TMGGAAGGCCGGTCCTAGGAGATGCTGGGGCTTCCATGAACCCACAGGTT
ST1	TAGGAAGGCCGGTCCTAGGAGATGCTGGGGCTTCCATGAACCCACAGGTT
DH1	TAGGAAGGCCGGTCCTAGGAGATGCTGGGGCTTCCATGAACCCACAGGTT
NF2	TAGGAAGGCCGGTCCTAGGAGATGCTGGGGCTTCCATGAACCCACMGGTT
NF1	TAGGAAGGCCGGTCCTAGGAGATGCTGGGGCTTCCMTGAACCCMCMGGTT

NF3	TAGGAAGGCCSGTCCTAGGAGATGCTGGGGCTTCCATGAACCCMCAGGTT
NF5	TAGGAAGGCCGGTCCTAGGAGATGCTGGGGCTTCCATGAACCCACAGGTT
ST4	TAGGAAGGCCGGTCCTAGGAGATGCTGGGGCTTCCATGAACCCACAGGTT
ST5	TAGGAAGGCCSGTCYTAGGAGATGCTGGGGCTTCCATGAACCCMCMGGTT
NF4	TAGGAAGGCCGGTCCTAGGAGATGCTGGGGCTTCCATGAACCCACAGGTT
DH2	TAGGAAGGCCGGTCCTAGGAGATGCTGGGGCTTCCATGAACCCACAGGTT
DH3	TAGGAAGGCCGGTCCTAGGAGATGCTGGGGCTTCCATGAACCCACAGGTT
DH4	TAGGAAGGCCGGTCCTAGGAGATGCTGGGGCTTCCATGAACCCACAGGTT
DH5	TAGGAAGGCCGGTCCTAGGAGATGCTGGGGCTTCCATGAACCCACAGGTT

PS	CTCATGGCATATCTGAGCCAAGGACAGAAAGACCAGGACCTCACCTGGTC
IW1	CTCATGGCATTCRTGAGCCAVGRACAGAAAGACCAGGACCBVABCHGSTY
IW2	CTCATGGCATATCTGAGCCAAGGACAGAAAGACCAGGAYCTCAMCKGGTC
IW3	CTCATGGCATATCTGAGCCAAGGACAGAAAGACCAGGYMKGCAMCTGGTC
IW4	CTCATGGCATATCHGAGCCARGGACAKAAAGACCAGGACCTVRCCTGGTC
ST3	CTCATGGCATATCTGAGCCAAGGACAGAAAGACCAGGRCCTCACCBKSTC
IW5	CTCATGGCATATCTGAGCCAAGGACAGAAAGACCAGGACCTHACCHGGTC
ST2	CTCATGGCATATCTGAGCCAAGGACAGAAAGACCAGGACCTCMCCTGGTC
ST1	CTCATGGCATATCTGAGCCAAGGACAGAAAGACCAGGACCTCDCHTSSTC
DH1	CTCATGGCATTCTCGAGCCAAGGACAGAAAGACCAGGACCTCACCTGGTC
NF2	CYCATGGCATTCMYGAGCCAAGGACAGAAARACCMGGACCTCACCTGGTC
NF1	YTCMTGGCATATCTGAGCCMAGGACAGAAAGACCAGGACCTCACYTKGTC
NF3	CTCATGGCATATCTGAGCCMAGGACAGAAAGACCAGGACCTCACCTGGTC
NF5	CTCATGGCATATCTGAGCCAAGGACAGAAAGACCAGGACCYCACCTGGTC
ST4	CTCATGGCATWTVTGAGCCAAGGACAGAAAGACCAGGACCYCMCCTGGTC
ST5	CTCMTGGCMTATMTGAGCCAMGGACAGAAAGACCAGGACCTCACCTGGTC
NF4	CTCMTGGCATHTCTGAGCCMAGGACAGAMAGACCAGGACCTCACCTGGTC
DH2	CTCATGGCATHWCTGAGCCAAGGACAGAAAGACCAGGACCTCCMCTGGYC
DH3	CTCATGGCATATCTGAGCCAAGGACAGAAAGACCAGGACCTCACCTGGTC

```
DH4           CTCATGGCATATCTGAGCCAAGGACAGAAAGACCAGGACCYCMCCTGGTC
DH5           CTCATGGCATATCTGAGCCAAGGACAGAAAGACCAGGACCYCACCTGGTC

PS            AGCACGTGGTCATCCCCTCCCTGCTCCTGTGGGACCTCACCCCCATGGTC
IW1           HGCACGTGGTCMTCCCCYCCCTGCTCCTGTGGGACCTCMCCCCCATGGYC
IW2           AGCCTKTCARHAKCCCCYVMMTGMDHCTGDDGGACCTCAHCCCCATGGTC
IW3           WGBCCGTGGTCATCCYCKCCCTGCTCCTGTGGGACCTCACCCCCATGGTC
IW4           AGBACVTGGTCATCMCCHCCMHGCTCSTSTGGGACCTCACYCYCATGYYG
ST3           AGYMCGWGGYCATCCMMTCRCCYGCDCCKGKGGACCTCACCCCCATGGTC
IW5           AGSABGTGGKTCBCCWCCCCTVCTTCCCWDGGBACCTCACCCSCATSGTC
ST2           AGCACGTGGTCATCCCCTCCCTGCTCCTGWGGRACCTCACCCCCATGGTC
ST1           MGCACGTGGTCATCCCCTCCCTGCTCCTGRGGGACCTCACCCCCATGGTC
DH1           AGCMCGTGGTCATCCCCTCCCTGCTCCTGTGGRACCTCACCCCCMTGGTC
NF2           MGCACGTGGTCATCCCCTCCCTGCTCCTGWGGGACCTCACCCCCMTGGTC
NF1           VGCACGTGGTBATCHCCYCCCYSCHCCTGTGGGACCTCACCCCCATGGTC
NF3           AGCACGTGGTCATCCCCTCCCTGCTCCTGTGGGACCTCACCCCCATGGTC
NF5           AGCACGTGGTCATCCCCTCCCTGCTCCTGTGGGACCTCACCCCCATGGTC
ST4           AGCMCGTGGTCMTCCCCYCCCTGCYCCTGTGGAACCTCACCCCCMTGGTC
ST5           AGCACGTGGTCATCCCCTCCCTGCTCCTGTGGGACCTCACCCCCATGGTC
NF4           AGCACGTGGTCATCCCCTCCCTGCTCCTGTGGGACCTCAMCCCCATGGTC
DH2           ASCACGTGGTCATCCCCTCCCTGCTCCTGTGGRACCTCACCCCCATGGTC
DH3           AGCACGTGGTCATCCCCTCCCTGCTCCTGTGGRACCTCACCCCCATGGTC
DH4           ARCMCGTGGTCATCCCCTCCCTGCTCCYGTGGRACCTCACCCCCATGGTC
DH5           AGCACGTGGTCATCCCCTCCCTGCTCCTGTGGRACCTCACCCCCMTGGTC

PS            AGCACCCCTGTCCCCTCCCTGTAGGCCCCCACCCCTGTGGTCAGCACCCC
IW1           MGCACCCCTGYCCCMTCCCYGTAGSCCCCCMCCCCTGKGGTCAGCACCCC
IW2           AGCACCCCTGTCCCMTCCCTGTAGGCCCCCACCCCTGTGGTCAGCACCCC
IW3           AGCACCCCTGTCCMMTCCCTGTAGGCCCCCACCCCTGTGGTCAGCACCCC
```

IW4	CACCCRCATGTCCSATCCCTSYAGACCCCASCCHTCGYRGTYAGCACCCC
ST3	AGCACCCCTGTCCCMTCCCTGTAGGCCCCYACCCCTGTGGTCAGCACCCC
IW5	AGYWCCCSKGTCCCMTCCCTGTAGGCCCCYACCCCTGTGGTCAGCACCCC
ST2	AGCACCCCTGTCCCMTCCCTGTAGGCCCCCACCCCTGTGGTCAGCACCCC
ST1	AGCACCCCTGTCCCMTCCCTGTAGGCCCCYACCCCTGTGGTCAGCACCCC
DH1	AGCACCCCTGTCCMMTCCCTGTAGGCCCCCACCCCTGTGGTCAGYACCCC
NF2	AGCACCCCTGTCCCMTCCCTGTAGGCCCCCMCCCCTGTGGTCMSCMCCCC
NF1	AGCACCCCTGTCCCMTCCCTGTAGGCCCCYACCCCTGTGGYCAGCACCCC
NF3	AGCACCCCTGTCCCMTCCCTGTAGGCCCCYACCCCTGTGGTCAGCACCCC
NF5	AGCACCCCTGTCCCMTCCCTGTAGGCCCCCACCCCTGTGGTCAGCACCCC
ST4	AGCACCCCYGTCCCMTCCCTGTAGGCCCCCMCCCCTGTGGTCMGCACCCC
ST5	AGCACCCCTGTCCCMTCCCTGTAGGCCCCTACCCCTGTGGTCAGCACCCC
NF4	AGCACCCCTGTCCMMTCCCTGTAGGCCCCYACCCCTGTGGTCASCACCCC
DH2	AGCACCCCTGTCCCCWCCCTGTAGGCCCCYACCCCTGTGGTCAGCACCCC
DH3	AGCACCCCTGTCCCMTCCCTGTAGGCCCCYACCCCTGTGGTCAGCACCCC
DH4	AGCACCCCTGTCCCMTCCCTGYARGCCCCYACCCCTGTGGTCMSCACCCC
DH5	AGCACCCCTGTCCCMTCCCTGTAGGCCCCCACCCCTGKGGTCAGCACCCC

PS	TTTCTGCAGGACTTTGTTCCTATGGTTAGCGTCCTGTGTGTTTCCTCGGG
IW1	TTTCYGCAGSACTTTGTTCCTATGSTTAGCSTCCTGTGTRTTYCCTCGGG
IW2	TTTCTGCAGGACTTTGTTCCTATGGTTAGCGTCCWGTGTGTTTCCTCGGG
IW3	TTTCTGCAGGACTTTGTTCCTATGGTTAGCGTCCWGTGTGTTTCCTCGGG
IW4	TTTCYGMASGACTTTGTTCCTATGGTTAGCGTCCTGTGTGTTTCCTCGGG
ST3	TTTCTGCAGGACTTTGTTCCTATGGTTAGCGTCCTGTGTGTTTCCTCGGG
IW5	TTTCTGCAGGACTTTGTTCCTATGGTTAGCGTCCTGTGTGTTTCCTCGGG
ST2	TTTCTGCAGGACTTTGTTCCTATGGTTAGCGTCCTGTGTGTTTCCTCGGG
ST1	TTTCTGCAGGACTTTGTTCCTATGGTTAGCGTCCTGTGTGTTTCCTCGGG
DH1	TTTCTGCAGGACTTTGTTCCTATGGTTAGMGTCCTGTGTGTTTCCTCGGG
NF2	TTTCTGCMSGACYTTGTTCCTATGGTTASCGTCCWGTGTGTTTCCTCGGG

```
NF1        TTTCYGCMSGACTTTGTTCCTATGGTTAGCGTCCWGTGTGTTTCCTCGGG
NF3        TTTCTGCASGACTTTGTTCCTATGGTTAGCGTCCTGTGTGTTTCCTCRRG
NF5        TTTCTGCAGGACTTTGTTCCTATGGTTAGCGTCCTGWGWRWTTCCTCGGG
ST4        TTYCTGCAGGACTTTGTTCCTATGGTTAGCGYCCTGTGTRTTTCCYCGGG
ST5        TTTCTGCAGGACTTTGTTCCTATGGTTAGCGTCCTGTGTGTTTCCTCGGG
NF4        TTTCTGCAGGACTTTGTTCCTAWGGTTAGCGTCCTGTGTGTTTCCTCGGG
DH2        TTTCTGCAGGACTTTGTTCCTATGGTTAGCGTCCTGTGYGTTTCCTCKGG
DH3        TTTCTGCASGACTTTGTTCCTATGGTTAGCGTCCTGTGTGTTTCCTCGGG
DH4        TTTCYGCMSGACTTWGTYCCYMTSGTTAGCGTCCTGTGYMWWWCCTCGGG
DH5        TTTCTGCASGACTTTGTTCCTATGGTTAGCGTCCTGTGWRTTTCCTCGGG

PS         GTGCCGGGTGCTCCTGATTCCCAGGGTGTCATAGCGCCCAGTGTGGTCCT
IW1        GTGCCGGGTGCTCCTGATTCCCAGGGTGTCATAGCGCCCAGTGTGGTCCT
IW2        GTGCCGGGTGCTCCTGATTCCCAGGGTGTCATAGCGCCCAGTGTGGTCCT
IW3        GTGCCGGGTGCTCCTGATTCCCAGGGTGTCATAGCGCCCAGTGTGGTCCT
IW4        GTGCCGGGTGCTCCTGATTCCCAGGGTGTCATAGCGCCCAGTGTGGTCCT
ST3        GTGCCGGGTGCTCCTGATTCCCAGGGTGTCATAGCGCCCAGTGTGGTCCT
IW5        GTGCCGGGTGCTCCTGATTCCCAGGGTGTCATAGCGCCCAGTGTGGTCCT
ST2        GTGCCGGGTGCTCCTGATTCCCAGGGTGTCATAGCGCCCAGTGTGGTCCT
ST1        GTGCCGGGTGCTCCTGATTCCCAGGGTGTCATAGCGCCCAGTGTGGTCCT
DH1        GTGCCGGGTGCTCCTGATTCCCAGGGTGTCATAGCGCCCAGTGTGGTCCT
NF2        GTGCCGGGTGCTCCTGATTCCCAGGGTGTCATAGCGCCCAGTGTGGTCCT
NF1        GTGCCGGGTGCTCCTGATTCCCAGGGTGTCATAGCGCCCAGTGTGGTCCT
NF3        GTGCCGGGTGCTCCTGATTCCCAGGGTGTCATAGCGCCCAGTGTGGTCCT
NF5        GTGCCGGGTGCTCCTGATTCCCAGGGTGTCATAGCGCCCAGTGTGGTCCT
ST4        GTGCCGGGTGCTCCTGATTCCCAGGGTGTCATAGCGCCCAGTGTGGTCCT
ST5        GTGCCGGGTGCTCCTGATTCCCAGGGTGTCATAGCGCCCAGTGTGGTCCT
NF4        GTGCCGGGTGCTCCTGATTCCCAGGGTGTCATAGCGCCCAGTGTGGTCCT
DH2        RTGCCGGGTGCTCCTGATTCMCAGGGTGTCATAGCGCCCAGTGTGGTCMT
```

DH3	GTGCCGGGTGCTCCTGATTCCCAGGGTGTCATAGCGCCCAGTGTGGTCCT
DH4	GTGCCGGGTGCTCCTGATTCCCAGGGTGTCATAGCGCCCAGTGTGGTCCT
DH5	GTGCCGGGTGCTCCTGATTCCCAGGGTGTCATAGCGCCCAGTGTGGTCCT

PS	TGGTCGGGCGGATGCACCACAAACACATGAACATTCTGTCAGAATGGGCG
IW1	TGGTCGGGCGGATGCACCACAAACACATGAACATTCTGTCAGAATGGGCG
IW2	TGGTCGGGCGGATGCACCACAAACACATGAACATTCTGTCAGAATGGGCG
IW3	TGGTCGGGCGGATGCACCACAAACACATGAACATTCTGTCAGAATGGGCG
IW4	TGGTCGGGCGGATGCACCACAAACACATGAACATTCTGTCAGAATGGGCG
ST3	TGGTCGGGCGGATGCACCACAAACACATGAACATTCTGTCAGAATGGGCG
IW5	TGGTCGGGCGGATGCACCACAAACACATGAACATTCTGTCAGAATGGGCG
ST2	TGGTCGGGCGGATGCACCACAAACACATGAACATTCTGTCAGAATGGGCG
ST1	TGGTCGGGCGGATGCACCACAAACACATGAACATTCTGTCAGAATGGGCG
DH1	TGGTCGGGCGGATGCACCACAAACACATGAACATTCTGTCAGAVKGDGCG
NF2	TGGTCGGGCGGATGCACCACAAACACATGAACATTCTGTCAGAATGGGCG
NF1	TGGTCGGGCGGATGCACCACAAACACATGAACATTCTGTCAGAATGGGCG
NF3	TGGTCGGGCGGATGCACCACAAACACATGAACATTCTGTCAGAATGGGCG
NF5	TGGTCGGGCGGATGCACCACAAACACATGAACATTYTKTCASAABGGSCV
ST4	TGGTCGGGCGGATGCACCACAAACACATGAACATTCTGTCAGAATGGGCG
ST5	TGGTCGGGCGGATGCACCACAAACACATGAACATTCTGTCAGAATGGGCG
NF4	TGGTCGGGCGGATGCACCACAAACACATGAACAYHCHGTVHGAATGGGBG
DH2	TGGTCGGGCGGATGCACCACAAACACATGAACATTCTGTCARAATGGGCG
DH3	TGGTCGGGCGGATGCACCACAAACACATGAACATTYTSTCASAATGGSCG
DH4	TGSYCGKKSGGATGCACCACAMACAHATGAACATTCTGTCAGAATGGGCG
DH5	TGGTCGGGCGGATGCACCACAAACACATGAACATTCTGTCAGAATGGGCG

PS	CTGCTTTCGCCCCCATGTTCAGCTCTCAGCTGCTGGACTGGACTTCTGGG
IW1	CTGCTTTCGCCCCCATGTTCAGCTCTCAGCTGCTGGACTGGACTTCTGGG
IW2	CTGCTTTCGCCCCCATGTTCAGCTCTCAGCTGCTGGACTGGACTTCTGGG

IW3 CTGCTTTCGCCCCCATGTTCAGCTCTCAGCTGCTGGACTGGACTTCTGGG
IW4 CTGCTTTCGCCCCCATGTTCAGCTCTCAGCTGCTGGACTGGACTTCTGGG
ST3 CTGCTTTCGCCCCCATGTTCAGCTCTCAGCTGCTGGACTGGACTTCTGGG
IW5 CTGCTTTCSCCCCCATGTTCAGCTCTCAGCTGCTGGACTGGACTTCTGGG
ST2 CTGCTTTCGCCCCCATGTTCAGCTCTCAGCTGCTGGACTGGACTTCTGGG
ST1 CTGCTTTCSCCCCCATGTTCAGCTCTCAGCTGCTGGACTGGACTTCTGGG
DH1 BBGCTTTYGCBCBHATGTWHAGCTCTCAGCTGCTGGACTGGACTTCTGGG
NF2 CTGCTTTCGCCCCCATGTTCAGCTCTCAGCTGCTGGACTGGACTWCTGGG
NF1 CTGCTTTCGCCCCCATGTTCAGCTCTCAGCTGCTGGACTGGACTWCTGGG
NF3 CTGCTTTCGCCCCCATGTTCAGCTCTCAGCTGCTGGACTGGACTWCTGGG
NF5 CTGVBTTVDVBCBCATGTWCAGCTCTCAGCTGCTGGACTGGACTTCTGGG
ST4 CTGCTTTCGCCCCCATGTTCAGCTCTCAGCTGCTGGACTGGACTTCTGGG
ST5 CTGCTTTCGCCCCCATGTTCAGCTCTCAGCTGCTGGACTGGACTTCTGGG
NF4 HTGCTTTCDBCMCCATGTTCAGCTCTCAGCTGCTGGACTGGACTTCTGGG
DH2 CTGCTTTCSCCCCCATGTTCAGCTCTCAGCTGCTGGACTGGACTTCTGGG
DH3 HTGCTKDYGMCCCYDTGTTVARYTCAYAGCTDCTGGACTGGACTTCTGGG
DH4 CTGCTTTCGCCCCCATGTTCAGCTCTCAGCTGCTGGACTGGACTTCTGGG
DH5 CTGCTTTCGCCCCCATGTTCAGCTCTCAGCTGCTGGACTGGACTTCTGGG

PS GTGTAGATTCGAGCTTCTTCAGGTCTTGGCCTCCATCCACGTGTTTTCTG
IW1 GTGTAGATTCGAGCTTCTTCAGGTCTTGGCCTCCATCCACGTGTTTTCTG
IW2 GTGTAGATTCGAGCTTCTTCAGGTCTTGGCCTCCRTYCRCSTGWTTTCTG
IW3 GTGTAGATTCGAGCTTCTTCAGGTCTTGGCCTCCRTCCACGTGTTTTCTG
IW4 GTGTAGATTCGAGCTTCTTCAGGTCTTGGCCTCCATCCACGTGTTTTCTG
ST3 GTGTAGATTCGAGCTTCTTCAGGTCTTGGCCTCCRTCCACGTGTTTTCTG
IW5 GTGTAGATTCGAGCTTCTTCAGGTCTTGGCCTCCRTCCACGTGTTWKMYK
ST2 GTGTAGATTCGAGCTTCTTCAGGTCTTGGCCTCCRTCCACGTGTTTTCTG
ST1 GTGTAGATTCGAGCTTCTTCAGGTCTTGGCCTCCATMYACGTGTTTTCTG
DH1 GTGTAGATTCGAGCTTCTTCAGGTCTTGGCCTCCRTCCACGTGTTTTCTG

NF2	GTGTAGATTCGAGCTTCTTCAGGTCTTGGCCTCCATYMMYKYGWBWTCTG
NF1	GTGTAGATTCGAGCTTCTTCAGGTCTTGGCCTCCATCCACGTGTTTTCTG
NF3	GTGTAGATTCGAGCTTCTTCAGGTCTTGGCCTCCATCYASDYGWTWDCTG
NF5	GTGTAGATTCGAGCTTCTTCAGGTCTTGGCCTCCATYMACKYGWTTDCTG
ST4	GTGTAGATTCGAGCTTCTTCAGGTCTTGGCCTCCGTCCACGTGTWKDCWR
ST5	GTGTAGATTCGAGCTTCTTCAGGTCTTGGCCTCCATCCACGTGTTTTCTG
NF4	GTGTAGATTCGAGCTTCTTCAGGTCTTGGCCTCCATCYASRYGWTTKCTG
DH2	GTGTAGATTCGAGCTTCTTCAGGTCTTGGCCTCCRTCCACGTGTTTTCTG
DH3	GTGTAGATTCGAGCTTCTTCAGGTCTTGGCCTCCRTYMRHDYGWTTDCTG
DH4	GTGTAGATTCGAGCTTCTTCAGGTCTTGGCCTCCRTCCACGTGTTTTCTG
DH5	GTGTAGATTCGAGCTTCTTCAGGTCTTGGCCTCCRTCCACGTGTTTTCTG

PS	CCTCTGACCACCCGTTAAGCCTATTTCCTGAATTCTTGGGTCAGCATTCC
IW1	CCTCTGACCACCCGTTAAGCCTATTTCCTGAATTCTTGGGTCAGCATTCC
IW2	CHTCTGWCSWCCMGTTAMSCCHWTDTCSTDAATTCTTDGKTYAKVDTTCC
IW3	CCTCTGACCACCCGTTAAGCCTATTTCCTGAATTCTTGGGTCAGCATTCC
IW4	CCTCTGACCACCCGTTAAGCCTATTTCCTGAATTCTTGGGTCAGCATTCC
ST3	CCTCTGACCACCCGTTAAGCCTATTTCCTGAATTCTTGGGTCAGCATTCC
IW5	MCTCTRMSYMMSCSWKHHRCCTHYTTCSTGAATTCTTGGGTYAKCATTYC
ST2	CCTCTGACCACCCGTTAAGCCTATTTCCTGAATTCTTGGGTCAGCATTCC
ST1	CCTCTGWCCACCCGTTAAGCCTATTTCCTGAATTCTTGGGTCAGCATTCC
DH1	CCTCTGACCACCCGTTAAGCCTATTTCCTGAATTCTTGGGTCAGCATTCC
NF2	CCTCTGACCACCCGTTAAGCCTATTTCCTGAATBCTTVGGTCVKCATTCC
NF1	CCTCTGACCACCCGTTAAGCCTATTTCCTGAATTCTTGGGTCAGCATTCC
NF3	CCTCTGWBSACCCRTTDHRCHTATTTVCTGAATTCTTGGGTCVKCATTCC
NF5	CYTCTGWCHACCCGTTAMGCCTATTTCCTGAATTCTTGGGTCAGCATTCC
ST4	MMWMWRAMYMCCCDDTHASCBTATTTCCTDAATTCTTGGKTCAGCATTCC
ST5	CCTCTGACCACCCGTTAAGCCTATTTCCTGAATTCTTGGGTCAGCATTCC
NF4	CCTCTGWCCACCCGTTAAGCCTATTTCCTGAATBCTTGGGTCVGCATTCC

```
DH2        CCTCTGACCACCCGTTAAGCCTATTTCCTGAATTCTTGGGTCAGCATTCC
DH3        CCTCTGWCCASYCRKTWAGCCTATTTCCTGAATTCTTGGGTCAGCATTCC
DH4        CCTCTGACCACCCGTTAAGCCTATTTCCTGAATTCTTGGGTCAGCATTCC
DH5        CCTCTGACCACCCGTTAAGCCTATTTCCTGAATTCTTGGGTCAGCATTCC

PS         CATTCTCCCATCTTGGAAATCCCACGTGGGTTTTCTCACAGGCTCCAGGT
IW1        CATTCTCCCATCTTGGAAATCCCACGTGGGTTTTCTCACAGGCTCCAGGT
IW2        CATTCTCCCATCTTGGAAATCCCACGTGGGTTTTCTCACAGGCTCCAGGT
IW3        CATTCTCCCATCTTGGAAATCCCACGTGGGTTTTCTCACAGGCTCCAGGT
IW4        CATTCTCCCATCTTGGAAATCCCACGTGGGTTTTCTCACAGGCTCCAGGT
ST3        CATTCTCCCATCTTGGAAATCCCACGTGGGTTTTCTCACAGGCTCCAGGT
IW5        CATTCTCCCATCTTGGAAATCCCACGTGGGTTTTCTCACAGGCTCCAGGT
ST2        CATTCTCCCATCTTGGAAATCCCACGTGGGTTTTCTCACAGGCTCCAGGT
ST1        CATTCTCCCATCTTGGAAATCCCACGTGGGTTTTCTCACAGGCTCCAGGT
DH1        CATTCTCCCATCTTGGAAATCCCACGTGGGTTTTCTCACAGGCTCCAGGT
NF2        CATTCTCCCATCTTGGAAATCCCACGTGGGTTTTCTCACAGGCTCCAGGT
NF1        CATTCTCCCATCTTGGAAATCCCACGTGGGTTTTCTCACAGGCTCCAGGT
NF3        CATTCTCCCATCTTGGAAATCCCACGTGGGTTTTCTCACAGGCTCCAGGT
NF5        CATTCTCCCATCTTGGAAATCCCACGTGGGTTTTCTCACAGGCTCCAGGT
ST4        CATTCTCCCATCTTGGAAATCCCACGTGGGTTTTCTCACAGGCTCCAGGT
ST5        CATTCTCCCATCTTGGAAATCCCACGTGGGTTTTCTCACAGGCTCCAGGT
NF4        CATTCTCCCATCTTGGAAATCCCACGTGGGTTTTCTCACAGGCTCCAGGT
DH2        CATTCTCCCATCTTGGAAATCCCACGTGGGTTTTCTCACAGGCTCCAGGT
DH3        CATTCTCCCATCTTGGAAATCCCACGTGGGTTTTCTCACAGGCTCCAGGT
DH4        CATTCTCCCATCTTGGAAATCCCACGTGGGTTTTCTCACAGGCTCCAGGT
DH5        CATTCTCCCATCTTGGAAATCCCACGTGGGTTTTCTCACAGGCTCCAGGT

PS         CTCTGGTTCGCAAGACTGCCCCATGGCAAGCCCAGCCGGAGCCACAGACC
IW1        CTCTGGTTCGMWMGACTGCMCCATGRCAAGCCCAGCCGGAGCCACAGACC
```

IW2	CTCTGGTTCGMYMGACTGCMCCATGRYAAGCCCAGCCGGAGCCACAGACC
IW3	CTCTGGTTCGCYAGACTGCCCCATGRCAAGCCCAGCCGGAGCCACAGACC
IW4	CTYWGGTTCGCAAGACTGCCCCATGGCAAGCCCAGCCGGAGCCACAGACC
ST3	CTCTGGTTCGCAAGACTGCCCCATGGCAAGCCCAGCCGGAGCCACAGACC
IW5	CTCTGGTTCGCAAGACTGCCCCATGGCASWMKCAKCMGGAGCCACARACC
ST2	CTCTGGTTCGCAAGACTGCCCCATGGCAMDSCCAGCCGGAGCCACAGACC
ST1	CTCTGGTTCGCAAGACTGCCCCATGGCAAGCCCAGCCGGAGCCACAGACC
DH1	CTCTGGTTCGCAAGACTGCCCCATGGCAAGCCCAGCCGGAGCCACAGACC
NF2	CTCTGGTTCGCACAGCTGCTCTYSGSSMAGCCCAGCCGGAGCCACAGACC
NF1	CTYWGGTTCGCAAGACTGCCCCATGGCAAGCCCAGCCGGAGCCACAGACC
NF3	CTCTGGTTCGCAAGACTGCCCCATGGCACWGSCWKCTSGGWSYCKRGACY
NF5	CTCTGGTTCGCAAGACTGCCCCATGGCAAGCCCAGCCGGAGCCACAGACC
ST4	CTCTGGTTCGCAWGACTGCCCCATGGCAAGCCCAGCCGGAGCCACAGACC
ST5	CTCTGGTTCGCAAGACTGCCCCATGGCAAGCCCAGCCGGAGCCACAGACC
NF4	CTCTGGTTCGCAAGACTGCCCCATGGCAAGCCCAGCCGGAGCCACAGACC
DH2	CWCTGGTTCGCAAGACTGCCCCATGRCAAGCCCAGCCGGAGCCACAGACC
DH3	CTCTGGTTCGCAAGACTGCCCCATGGCAAGCCCAGCCGGAGCCACAGACC
DH4	CTCTGGTTCGCAAGACTGCCCCATGGCAAGCCCAGCCGGAGCCACAGACC
DH5	CTCTGGTTCGCAAGACTGCCCCATGGCAAGCCCAGCCGGAGCCACAGACC

PS	ACCTGGTGTGGCCTCCAGCGCAGCGCCCCTGCCCGTGTGCGTGCCGCCCT
IW1	ACCHGGTGTGGCCTCCAGCGCAGCGCCCCTGCCCGTGTGCGTGCCGCCCT
IW2	ACCTGGTGTGGCCTCCAGCGCAGCGCCCCTGCCCGTGTGCGTGCCGCCCT
IW3	ACCTGGTGTGGCCTCCAGCGCAGCGCCCCTGCCCGTGTGCGTGCCGCCCT
IW4	ACCTGGTGTGGCCTCCAGCGCAGCGCCCCTGCCCGTGTGCGTGCCGCCCT
ST3	ACCTGGTGTGGCCTCCAGCGCAGCGCCCCTGCCCGTGTGCGTGCCGCCCT
IW5	AYMWRRWGTGGCCTCCAGCGCARCGCCCCTGCCCGTGTGCGTGCCGCCCT
ST2	ACCTGGTGTGGCCTCCAGCGCAGCGCCCCTGCCCGTGTGCGTGCCGCCCT
ST1	ACCWGGTGTGGCCTCCAGCGCAGCGCCCCTGCCCGTGTGCGTGCCGCCCT

DH1	ACCTGGTGTGGCCTCCAGCGCAGCGCCCCTGCCCGTGTGCGTGCCGCCCT
NF2	ACCTGGTGTGGCCTCCAGCGCAGCGCCCCTGCCCGTGTGCGTGCCGCCCT
NF1	ACCTGGTGTGGCCTCCAGCGCAGCGCCCCTGCCCGTGTGCGTGCCGCCCT
NF3	AYMWRRTGWGGCCWCCAGCGCAGCGCCCCTGMCCGTGTGCGTGCCGCMCT
NF5	ACCTGGTGTGGCCTCCAGCGCAGCGCCCCTGCCCGTGTGCGTGCCGCCCT
ST4	ACCTGGTGTGGCCTCCAGCGCAGCGCCCCTGCCCGTGTGCGTGCCGCCCT
ST5	ACCTGGTGTGGCCTCCAGCGCAGCGCCCCTGCCCGTGTGCGTGCCGCCCT
NF4	ACCTGGTGTGGCCTCCAGCGCAGCGCCCCTGCCCGTGTGCGTGCCGCCCT
DH2	ACCTGGTGTGGCCTCCAGCGCAGCGCCCCTGCCCGTGTGCGTGCCGCCCT
DH3	ACCTGGTGTGGCCTCCAGCGCAGCGCCCCTGCCCGTGTGCGTGCCGCCCT
DH4	AHCTGATGTGGCCTCCAGCGCAGCGCCCCTGCCCGTGTGCGTGCCGCCCT
DH5	ACCTGGTGTGGCCTCCAGCGCAGCGCCCCTGCCCGTGTGCGTGCCGCCCT

PS	GCCTGCCCACACGTCTGTGCCTTCTTCCAGAGCTCAGCCGGCAGCGAGGG
IW1	GCCTGCCCACACGTCTGTGCCTTCTTCCAGAGCTBAGCCGGCAGCGAGGG
IW2	GCCTGCCCACACGTCTGTGCCTTCTTCCAGAGCTCAGCCGGCAGCGAGGG
IW3	GCCTGCCCACACGTCTGTGCCTTCTTCCAGAGCTCAGCCGGCAGCGAGGG
IW4	GCCTGCCCACACGTCTGTGCCTTCTTCCAGAGCTCAGCCGGCAGCGAGGG
ST3	GCCTGCCCACACGTCTGTGCCTTCTTCCAGAGCTBAGCCGGCAGCGAGGG
IW5	GCCTGCCMACACRTCTGTGCCTTCTTCCAGAGCTCAGCCGGCAGCGAGGG
ST2	GCCTGCCCACACGTCTGTGCCTTCTTCCAGAGCTCAGCCGGCAGCGAGGG
ST1	GCCTGCCCACACGTCTGTGCCTTCTTCCAGAGCTMAGCCGGSAGCGAGGG
DH1	GCCTGCCCACACGTCTGTGCCTTCTTCCAGAGCTCAGCCGGCAGCGAGGG
NF2	GCCTGCCCACACGTCTGTGCCTTCTTCCAGAGCTCAGCCGGCAGCGAGGG
NF1	GCCTGCCCACACGTCTGTGCCTTCTTCCAGAGCTCAGCCGGCAGCGAGGG
NF3	GMCWGCCMACACGTCTGTGCCTTCTTCCAGAGCTCAGCCGGCAGCGAGGG
NF5	GCCTGCCCACACGTCTGTGCCTTCTTCCAGAGCWCWGGAGGCGGCGAGGG
ST4	GCCTGCCCACACGTCTGTGCCTTCTTCCAGAGCTBAGCCGGCAGCGAGGG
ST5	GCCTGCCCACACGTCTGTGCCTTCTTCCARAGCTBAGCCGGCAGCGAGGG

NF4 GCCTGCCCACACGTCTGTGCCTTCTTCCAGAGCTSGWCGAGCSGKGAGGG
DH2 GCCTGCCCACACGTCTGTGCCTTCTTCCAGAGCTMAGCCGGCAGCGAGGG
DH3 GCCTGCCCACACGTCTGTGCCTTCTTCCAGAGCTCAGCCGGCAGCGAGGG
DH4 GCCTGCCCACACGTCTGTGCCTTCTTCCAGAGCTMAGCCGGCAGCGAGGG
DH5 GCCTGCCCACACGTCTGTGCCTTCTTCCAGAGCTCAGCCGGCAGCGAGGG

PS CCCCATTCCCTCTGGGGCAGGAGTGTCCCGCTGTCGTAGCCCTGTGGAGG
IW1 CCCCATTCCCTCTGGGGCAGGAGTGTCCCGCTGTCGTWGCCCTGTGGARG
IW2 CCCCATTCCCTCTGGGGCAGGAGTGTCCAGCTGTCGTAGCCCTGTGGAGG
IW3 CCCCATTCCCTCTGGGGCAGGAGTGTCCAGCTGTCGTAGCCCTGTGGAGG
IW4 CCCCATTCCCTCTGGGGCAGGAGTGTCCMGCTGTCGTWGSCCTGTGGARG
ST3 CCCCATTCCCTCTGGGGCAGGAGTGTCCMGCTGTCGTAGSCCTGTGGARG
IW5 CCCCATTCCCTCTGGGGCAGGAGTGTCCCGYTGKCGCACCMSGRKAGAGG
ST2 CCCCATTCCCTCTGGGGCAGGAGTGTCCAGCTGTCGTAGCCCTGTGGAGG
ST1 CCCCATTCCCTCTGGGGCAGGAGTGTCCMGCBGTCGTAGSCCTGTGGARG
DH1 CCCCATTCCCTCTGGGGCAGGAGTGTCCAGCTGTCGTAGCCCTGTGGAGG
NF2 CCCCATTCCCTCTGGGGCAGGAGTGTCCAGCTGTCGTAGCCCTGTGGAGG
NF1 CCCCATTCCCTCTGGGGCAGGAGTGTCCAGCTGTCGTAGCCCTGTGGAGG
NF3 CCCCATTCCCTCTGGGGCASGAGTGTYSAGCTGYSGYAKGSCTRKGKAAG
NF5 CCCCATTCCCTCTGGGGCAGGAGTGTCCAGCTGTCGTAGCCCTGTGGAGG
ST4 CCCCATTCCCTCTGGGGCAGGAGTGTCCAGCTGTCGTAGCCCTGTGGAGG
ST5 CCCCATTCCCTCTGGGGCAGGAGTGTCCAGCTGTCGTAGCCCTGTGGAGG
NF4 CCCCATTCCCTCTGGGGCAGGAGTGTYSAGCTGTCGTAGSCCTGTGGARG
DH2 CCCCATTCCCTCTGGGGCAGGAGTGTCCAGCTGTCGTAGCCCTGTGGAGG
DH3 CCCCATTCCCTCTGGGGCAGGAGTGTCCAGCTGTCGTAGCCCTGTGGAGG
DH4 CCCCATTCCCTCTGGGGCAGGAGTGTCCAGCTGTCGTAGCCCTGTGGAGG
DH5 CCCCATTCCCTCYGGGGCAGGAGTGTCCAGCTGTCGTAGCCCTGTGGAGG

PS CCAGAGGGTCCTCCACCCATGTCGCGGGGATGCGCTCCAGCAACCACCCC

IW1	CCAGAGGGTCCTCCACCCATGTCGCGGGRATGCGCTCCAGCAACCAHCCS
IW2	CCARAGGGTCCTCCACCMAWGTCGCGGGGATGCGCTCCAGCAACCACCCC
IW3	CCAGAGGGTCCTCCACCCATGTCGCGGGGATGCGCTCCAGCAACCACCCC
IW4	CCAGAGGGTCYTCCACMCATGTCSCGGGRATGCGCTCCAGCAACCACCCC
ST3	CCAGAGGGTCCTCCACCCATGTCGCGGGRATGCGCTCCAGCAACCACCCC
IW5	SYMSATARTCCTCCACCCATGTCSCSGGGATGCGCTCCAGCAACCACCCC
ST2	CCAGAGGGTCCTCCACCMATGTCGCGGGRATGCGCTCCAGCAACCACCCC
ST1	CCAGAGGGTCYTCCACCCATGTSGCGGGGATGCGCTCCAGCAACCACCCC
DH1	CCAGAGGGTCCTCCACCCATGTCGCGGGRATGCGCTCCAGCAACSACVCS
NF2	CCAGAGGGTCCTCCACCCATGTCGCGGGGATGCGCTCCAGCAACCACMCC
NF1	CCAGAGGGTCCTCCACCCATGTCGCGGGGATGCGCTCCAGCAACCACMCC
NF3	YCAGAGGSYYYTCCASMSAKGTCGCGGGGATGCGCTCCAGCAACCACVCS
NF5	CCAGAGGGTCCTCCACCCATRTCGCGGGRATGCGCTCCAGCAASSRCCCV
ST4	CCAGAGGGTCCTCCACCCATWWTARGGGMRGGKGRTAGGTTCRMAGKYSY
ST5	CCAGAGGGTCCTCCACCCATGTCGCGGGRATGCGCTCCAGCAACCACCVC
NF4	CCAGAGGGTCCTCCACCCATGTCGCGGGGATGCGCTCCAGCAACCACVCV
DH2	CCAGAGGGTCCTCCACCCATGTHKSGGGGATGCGCTCCAGCAACCACCCC
DH3	CCAGAGGGTCCTCCACCCATGTHKSGGGGATGCGCTCCAGCAACCACCCC
DH4	CCAGAGGGTCCTCCACCCATGTCGCGGGGATGCGCTCCAGCAACCACCCC
DH5	CCAGAGGGTCCTCCACCCATGTCSCGGGGATGCGCTCCAGCAASSDCMCS

PS	TCCTTCTTCCTCCGTGTTATCGCGGACACCGCGTCGTGCTGCTACTCCCT
IW1	TCCTTCTTCCTCCGTGTTATCRCGGACACCGCGTCGTGCTGCTACTCCCT
IW2	TCCTTCTTCCTCCGTGTTATCACGGACACCGCGTCGTGCTGCTACTCCCT
IW3	TCCTTCTTCCTCCGTGTTATCACGGACACCGCGTCGTGCTGCTACTCCCT
IW4	TCCTTCTTCCTCCGTGTTATCRCGGACACCGCGTCGTGCTGCTACTCCCT
ST3	TCCTTCTTCCTCCGTGTTATCRCGGACACCGCGTCGTGCTGCTACTCCCT
IW5	TCCTTCTTCCTCCGTGTTATCRCGGACACCGCGTCGTGCTGCTACTCCCT
ST2	TCCTTCTTCCTCCGTGTTATCACGGACACCGCGTCGTGCTGCTACTCCCT

ST1	TCCTTCTTCCTCCGTGTTATCRCGGACACCGCGTCGTGSTGCTACTCCCT
DH1	TCVTTCTTCCTCCGTGTTATCACGGACACCGCGTCGTGCTGCTACTCCCT
NF2	TCCTTCTTCCTCCGTGTTATCACGGACACCGCGTCGTGCTGCTACTCCCT
NF1	TCCTTCTTCCTCCGTGTTATCACGGACACCGCGTCGTGCTGCTACTCCCT
NF3	TCCTTCTTCCTCCGTGTTATCGCGGACACCGCGTCGTGCTGCTACTCCCT
NF5	TCCTTCTTCCTCCGTGTTATCACGGACACCGCGTCGTGCTGCTACTCCCT
ST4	KMMWTMTKMCWMCRWGTWATSACGRACACCGCGTCGWGCTGCTMCTCCCT
ST5	TCCTTCTTCCTCCGTGTTATCGCGGACACCGCGTCGTGCTGCTACTCCCT
NF4	TVVTTCTTCCTCCGTGTTATCACGGACACCGCGTCGTGCTGCTACTCCCT
DH2	TCCTTCTTCCTCCGTGTTATCACGGACACCGCGTCGTGCTGCTACTCCCT
DH3	TCCTTCTTCCTCCGTGTTATCACGGACACCGCGTCGTGCTGCTACTCCCT
DH4	TCCTTCTTCCTCCGTGTTATCACGGACACCGCGTCGTGCTGCTACTCCCT
DH5	TCCTTCTTCCTCCGTGTTATCACGGACACCGCGTCGTGCTGCTACTCCCT

PS	TCTCAAAGCCAGGAACGCAGGTGTGCGTGGGTGCTAAGTAGGCGCCGCTG
IW1	TCTCAAAGCCAGGAACGCAGGTGTGCGTGGGTGCTAAGTAGGCGCCGCTG
IW2	TCTCAAAGCCAGGAACGCAGGTGTGCGTGCGTGCTAAGTAGGCGCCGCTG
IW3	TCTCAAAGCCAGGAACGCAGGTGTGCGTGSGTGCTAAGTAGGCGCCGCTG
IW4	TCTCAAAGCCAGGAACGCAGGKGTGCGTGGGTGCTAAGTAGGCGCCGCTG
ST3	TCTCAAAGCCAGGAACGCAGGTGTGCGTGGGTGCTAAGTAGGCGCCGCTG
IW5	TCTCAAAGCCAGGAACGCAGGKGTGCGTGSGTGCTAAGTAGGCGCCGCTG
ST2	TCTCAAAGCCAGGAACGCAGGTGTGCGTGGGTGCTAAGTAGGCGCCGCTG
ST1	TCTCAAAGCCAGGAACGCAGGKGTGCGTGGGTGCTAAGTAGGCGCCGCTG
DH1	TCTCAAAGCCAGGAACGCAGGTGTGCGTGSGTGCTAAGTAGGCGCCGCTG
NF2	TCTCAAAGCCAGGAACGCAGGTGTGCGTGGGTGCTAAGTAGGCGCCGCTG
NF1	TCTCAAAGCCAGGAACGCAGGTGTGCGTGGGTGCTAAGTAGGCGCCGCTG
NF3	TCTCAAAGCCAGGAACGCAGGTGTGCGTGGGTGCTAAGTAGGCGCCGCTG
NF5	TCTCAAAGCCAGGAACGCAGGTGTGCGTGGGTGCTAAGTAGGCGCCGCTG
ST4	TCTCAAAGCCAGGAACGMAGGTGTGCGTGGGTGCTAAGTAGGCGCCGCTG

ST5 TCTCAAAGCCAGGAACGCAGGTGTGCGTGGGTGCTAAGTAGGCGCCGCTG
NF4 TCTCAAAGCCAGGAACGCAGGTGTGCGTGGGTGCTAAGTAGGCGCCGCTG
DH2 TCTCAAAGCCAGGAACGCAGGTGTGCGTGCGTGCTAAGTAGGCGCCGCTG
DH3 TCTCAAAGCCAGGAACGCAGGTGTGCGTGCGTGCTAAGTAGGCGCCGCTG
DH4 TCTCAAAGCCAGGAACGCAGGTGTGCGTGCGTGCTAAGTAGGCGCCGCTG
DH5 TCTCAAAGCCAGGAACGCAGGTGTGCGTGSGTGCTAAGTAGGCGCCGCTG

PS GTGGGTTTGTGGAGTCACATGGGTGCTAGCCTCATCTCCCTCCAGCCCTG
IW1 GTGGGTTTGTGGAGTCACATGGGTGCTAGCCBMAYCTCCCTCCAGCCCTG
IW2 GTGGGTTTGTGGAGTCA-ATGGGTGCTAGCCTCATCTCCCTCCAGCCCTG
IW3 GTGGGTTTGTGGAGTCA-ATGGGTGCTAGCCTCATCTCCCTCCAGCCCTG
IW4 GTGGGTTTGTGGAGTCACATGGGTGCTAGCCTCATCTCCCTCCAGCCCTG
ST3 GTGGGTTTGTGGAGTCACATGGGTGCTAGCCTCATCTCCCTCCAGCCCTG
IW5 GTGGGTTTGTGGAGTCACATGGGTGCTAGCCTCATCTCCCTCCAGCCCTG
ST2 GTGGGTTTGTGGAGTCACATGGGTGCTAGCCTCATCTCCCTCCAGCCCTG
ST1 GKGGGTTTGTGGAGTCA-ATGGGTGCTAGCCTCATCTTCCTCCAGCCMTG
DH1 GTGGGTTTGTGGAGTCACATGGGTGCTAGCCTCATCWTCCTCCAGCCMWG
NF2 GTGGGTTTGTGGAGTCACATGGGTGCTAGCCTCATCTTSCTCCAGCCMWG
NF1 GTGGGTTTGTGGAGTCACATGGGTGCTAGCCTCATCTTSCTCCAGCCMWG
NF3 GTGGGTTTGTGGAGTCACATGGGTGCTAGCCTCATCYYRYTMCWGCCMWY
NF5 GTGGGTTTGTGGAGTCACATGGGTGCTAGCCTCATCTVCCTCCAGCCCTG
ST4 GTGGGTTTGTGGAGTCACATGGGTGCTAGCCTCATCTYCCTMCAGCCMTG
ST5 GTGGGTTTGTGGAGTCACATGGGTGCTAGCCTCATCTTCCTCCAGCCMWG
NF4 GTGGGTTTGTGGAGTCACATGGGTGCTAGCCTCATCSWMCTMCWKSCMTG
DH2 GTGGGTTTGTGGAGTCACATGGGTGCTAGCCTCATCTCCCTCCAGCCCTG
DH3 GTGGGTTTGTGGAGTCACATGGGTGCTAGCCTCATCTCCCTCCAGCCCTG
DH4 GTGGGTTTGTGGAGTCA-ATGGGTGCTRYRCTCYMCTCCCTCCAGCCCTG
DH5 GTGGGTTTGTGGAGTCACATGGGTGCTAGCCTCMTCTYCCTCCAGCCCTG

PS	CTGGTAGGTGTCCTCTATGCCCTGACCTGCATCCCCGAGCCCCTTCCCCG
IW1	CTGGTAGGTGTCCTCTATGVCCTGACCTGCATCCCCGAGCCCCTTCCCCG
IW2	CTGGTAGGTGTCCTCTATGCCCTGACCTGCATCCCCRAGCCCCTTCCCCG
IW3	CTGGTAGGTGTCCTCTATGCCCTGACCTGCATCCCCGAGCCCCTTCCCCG
IW4	CTGGTAGGTGTCCTCTATGCCCTGACCTGCATCCCCGAGCCCCTTCCCCG
ST3	CTGGTAGGTGTCCTCTATGCCCTGACCTGCATCCCCGAGCCCCTTCCCCG
IW5	CTGGTAGGTGTCCTCTATGCCCTGACCTGCATCCCCGAGCCCCTTCCCCG
ST2	CTGGTAGGTGTCCTCTATGCCCTGACCTGCATCCCCGAGCCCCTTCCCCG
ST1	MTGGTASGTGTCCTCTATGMCCWGACCTGCATCCCCGAGCCCCTTCCCCG
DH1	STGGTAGGTRTCCTCTATGCCCTGACCTGCATCCCCGAGCCCCTTCCCCG
NF2	STGGTAGGTRTCCTCTATGMCCTGASCTGCATCCCCGAGCCCCTTCCCYG
NF1	STGGTAGGTRTCCTCTATGMCCTGASCTGCATCCCCGAGCCCCTTCCCYG
NF3	RTGGYAGGTRWCCTCTATGMMCTGASCTGCATCCCCGAGSCCCTTCCCYK
NF5	CTGGTAGGTGTCCTCTATGCCCTGACCTGCATCCCCGAGCCCCTTCCCCG
ST4	CTGGTAGGTGTCCTCTATGVCCTGACCTGCATCCCCGAGCCCCTTCCCCG
ST5	CTGGTAGGTGTCCTCTATGCCCTGACCTGCATCCCCGAGCCCCTTCCCCG
NF4	STGGTAGGTRTCCTCTATGMCCTGACCTGCMTCCCCGAGCCCCTTCCCCG
DH2	CTGGTAGGTGDCCTCTATGCCCTGACCTGCATCCCCGAGCCCCTTCCCCG
DH3	CTGGTAGGTGDCCTCTATGCCCTGACCTGCATCCCCGAGCCCCTTCCCCG
DH4	VTGGTAGGTGTCCTCTATGCCCTGACCTGCATCCCCGAGCCCCTTCCCCG
DH5	CTGGTAGGTGTCCTCTATGCCCTGACCTGCATCCCCGAGCCCCTTCCCCG

PS	GCTGCCCCGACACCACCAGAGGGACCCTCAGCAGCAGATCTTCGCAGACC
IW1	GCTGCCCCGACACCACCAGAGGGACCCTCAGCAGCAGATCTTCGCAGACC
IW2	GCTGCCCCGACACCACCAGAGGGACCCTCAGCAGCAGATCTTCGCAGACC
IW3	GCTGCCCCGACACCACCAGAGGGACCCTCAGCAGCAGATCTTCGCAGACC
IW4	GCTGCCCCGACACCACCAGAGGGACCCTCAGCAGCAGATCTTCGCAGACC
ST3	GCTGCCCCGACACCACCAGAGGGACCCTCAGCAGCAGATCTTCSCAGACC
IW5	GYTGCCCCGACACCACCAGAGGGACCCTCAGCAGCAGATCTTCGCAGACC

ST2	GCTGCCCCGACACCACCAGAGGGACCCTCAGCAGCAGATCTTCGCAGACC
ST1	GYTGCCCCGACACCACSAGARGGACCCTCAGCAGCWGATCTTCSCRKACC
DH1	GCTGCCCCGACACCACCAGAGGGACCCTCAGCAGCAGATCTTCSCAGACC
NF2	GCTGCCCCGACACCACCARARGGACCCTCAGCAGCAGATCTTCSCAGACC
NF1	GCTGCCCCGACACCACCARARGGACCCTCAGCAGCAGATCTTCSCAGACC
NF3	GSTGCCCCGACACCACMARAKGGACCCTCAGCAGCMGWKCTTCSCAGACC
NF5	GCTGCCCCGACACCACCAGAGGGACCCTCAGCAGCAGATCTTCGCAGACC
ST4	GCTGCCCCGACACCACCAGAGGGACCCTCAGCAGCARATCTTCGCAGACC
ST5	GCTGCCCCGACACCACCAGARGGACCCTCAGCAGCAGATCTTCSCAGACC
NF4	GCTGCCCCGACACCACCAGARGGACCCTCAGCAGCAGATCTTCSCAGACC
DH2	GCTGCCCCGACACCACCAGAGGGACCCTCAGCAGCAGATCTTCSCAGACC
DH3	GCTGCCCCGACACCACCAGAGGGACCCTCAGCAGCAGATCTTCSCAGACC
DH4	GCTGCCCCGACACCACCAGAGGGACCCTCAGCAGCAGATCTTCGCAGACC
DH5	GCTGCCCCGACACCACCAGAGGGACCCTCAGCAGCAGATCTTCGCAGACC

PS	TCTCTCCCACCCATACCAGCTGGCACCTGGAGCCCCGTACCCGGGGCCCA
IW1	TCTCTCCCACCCATACCAGCTGGVACSTGGASCCCCGTACCCGGGGCCCA
IW2	TCTCTCCCACCCATACCAGCTGRCACCTGGAGCCCCGTACCCGGGGCCCA
IW3	TCTCTCCCACCCATACCAGCTGRCACCTGGASCCCCGTACCCGGGGCCCA
IW4	TCTCTCCCACCCATACCAGCTGRCACSTGGAGCCCCGTACCCGGGGCCCA
ST3	TCTCTCCCACCCATACCAGCTRRCACCTGGASCCCCGTACCCGGGGCCCA
IW5	TCTCTCCCACCCATACCAGCTGRCACSTGGAGCCCCGTACCCGGGGCCCA
ST2	TCTCTCCCACCCATACCAGCTGGCACCTGGAGCCCCGTACCCGGGGCCCA
ST1	TMTCTCCCACCCATACCAGCTGGCACSTGGAGCCCCGTACCCGGGGCCCA
DH1	TCTCTCCCACCCATACCAGCTGGCACCTGGAGCCCCGTACCCGGGGCCCA
NF2	TCTCTCCCACCCATACCAGCTGGCACCTGGAGCCCCGTACCCGGGGCCCA
NF1	TCTCTCCCACCCATACCAGCTGGCACCTGGAGCCCCGTACCCGGGGCCCA
NF3	TCTCTCCCACCCATACCAGCTGGCACCTGGAGCCCCGTACCCGGGGCCCA
NF5	TCTCTCCCACCCATACCAGCTGGCACCTGGAGCCCCGTACCCGGGGCCCA

ST4	TCTCTCCCACCCATACCAGCTGRMACCTGGAGCCCCGTACCCGGGGCCCA
ST5	TCTCTCCCACCCATACCAGCTGGCACCTGGAGCCCCGTACCCGGGGCCCA
NF4	TCTCTCCCACCCATACCAGCTGGCACCTGGAGCCCCGTACCCGGGGCCCA
DH2	TCTCTCCCACCCATACCADHHGGCACCTGGAGCCCCGTACCCGGGGCCCA
DH3	TCTCTCCCACCCATACCADHHGGCACCTGGAGCCCCGTACCCGGGGCCCA
DH4	TCTCTCCCACCCATACCAGCTGRCACCTGGAGCCCCGTACCCGGGGCCCA
DH5	TCTCTCCCACCCATACCAGCTGRCHCSTGGAGCCCCGTACCCGGGGCCCA

PS	GACCATGCCCACCTGGCCTTCTGCGGGGTCCCGAGGCCATCCTAGAGGCC
IW1	GACCATGCCCACCTGGCCTTCTGCGGGGTCCCGAGGCCATCCTAGAGGCC
IW2	GACCATGCCCACCTGGCCTTCTGCGGGGTCCCGAGGCCATCCTARAGGCC
IW3	GACCATGCCCACCTGGCCTTCTGCGGGGTCCCGAGGCCATCCTARAGGCC
IW4	GACCATGCCCACCTGGCCTTCTGCGGGGTCCCGAGGCCATCCTARAGGCC
ST3	GACCATGCCCACCTGGCCTTCTGCGGGGTCCCGAGGCCATCCTAGAGGCC
IW5	GACCATGCCCACCTGGCCTTCTGCGGGGTCCCGAGGCCATCCTARAGGCC
ST2	GACCATGCCCACCTGGCCTTCTGCGGGGTCCCGAGGCCATCCTAGAGGCC
ST1	GACCATGCCCACCTGGCCTTCTGCGGGGTCCCGAGGCCATCCTARAGGCC
DH1	GACCATGCCCACCTGGCCTTCTGCGGGGTCCCGAGGCCATCCTAGAGGCC
NF2	GACCATGCCCACCTGGCCTTCTGCGGGGTCCCGAGGCCATCCTARAGGCC
NF1	GACCATGCCCACCTGGCCTTCTGCGGGGTCCCGAGGCCATCCTARAGGCC
NF3	GACCATGCCCACCTGGCCTTCTGCGGGGTCCCGAGGCCATCCTARAGGCC
NF5	GACCATGCCCACCTGGCCTTCTGCGGGGTCCCGAGGCCATCCTAGAGGCC
ST4	RACCATGCCCACCTGGCCTTCTGSGGGGTCCCGAGGCCATCMTARAGGCC
ST5	GACCATGCCCACCTGGCCTTCTGCGGGGTCCCGAGGCCATCCTAGAGGCC
NF4	GACCATGCCCACCTGGCCTTCTGCGGGGTCCCGAGGCCATCCTAGAGGCC
DH2	GACCATGCCCACCTGGCCTTCTGCGGGGTCCCGAGGCCATCCTAGAGGCC
DH3	GACCATGCCCACCTGGCCTTCTGCGGGGTCCCGAGGCCATCCTAGAGGCC
DH4	GACCATGCCCACCTGGCCTTCTGCGGGGTCCCGAGGCCATCCTAGAGGCC
DH5	RACCATGCCCACCTGGCCTTCTGSGGGGTCCCGAGGYCATCYTARAGGCC

PS	ACTTTCCGGGGCATTCATGTATCACTCGTTCCCCTACAGCCAAGGCACAC
IW1	ACTTTCCGGGGCATTCATGTATCACTCGTTCCCCTACAGCCAAGGSACAC
IW2	ACTTTCCGGGGCATTCATGTATCACTCGTTCCCCTACAGCCAAGGCACAC
IW3	ACTTTCCGGGGCATTCATGTATCACTCGTTCCCCTACAGCCAAGGCACAC
IW4	ACTTTCCGGGGCATTCATGTATCACTCGTTCCCCTACAGCCAAGGSACAC
ST3	ACTTTCCGGGGCATTCATGTATCACTCGTTCCCCTACAGCCAAGGCACAC
IW5	ACTTTCCGGGGCATTCATGTATCACTCGTTCCCCTACAGCCAAGGSACAC
ST2	ACTTTCCGGGGCATTCATGTATCACTCGTTCCCCTACAGCCAAGGCACAC
ST1	ACTTTCCGGGGCATTCATGTATCACTCGTTCCCCTACAGCCAAGGCACAC
DH1	ACTTTCCGGGGCATTCATGTATCACTCGTTCCCCTACAGCCAAGGCACAC
NF2	ACTTTCCGGGGCATTCATGTATCACTCGTTCCCCTACAGCCAAGGCACAC
NF1	ACTTTCCGGGGCATTCATGTATCACTCGTTCCCCTACAGCCAAGGCACAC
NF3	ACTTTCCGGGGCATTCATGTATCACTCGTTCCCCTACAGCCAAGGCACAC
NF5	ACTTTCCGGGGCATTCATGTATCACTCGTTCCCCTACAGCCAAGGCACAC
ST4	ACTTTCCGGGGCATTCATGTATCACTCSTTCCCCTACAGCCAAGGSMCAC
ST5	ACTTTCCGGGGCATTCATGTATCACTCGTTCCCCTACAGCCAAGGCACAC
NF4	ACTTTCCGGGGCATTCATGTATCACTCGTTCCCCTACAGCCAAGGCACAC
DH2	ACTTTCCGGGGCATTCATGTATCACTCGTTCCCCTACAGCCAAGGCACAC
DH3	ACTTTCCGGGGCATTCATGTATCACTCGTTCCCCTACAGCCAAGGCACAC
DH4	ACTTTCCGGGGCATTCATGTATCACTCGTTCCCCTACAGCCAAGGCACAC
DH5	ACTTYCSGGGGCWTTCATGTATCACTCGTTCCCCTASAGCCAAGGSACAC

PS	CTGA
IW1	CTGA
IW2	CTRA
IW3	CTGA
IW4	CTGA
ST3	CTGA

```
IW5            CTGA
ST2            CTGA
ST1            CTGA
DH1            CTGA
NF2            CTGA
NF1            CTGA
NF3            CTGA
NF5            CTGA
ST4            CTRK
ST5            CTGA
NF4            CTGA
DH2            CTGA
DH3            CTGA
DH4            CTGA
DH5            CTRA
;
end;

#NEXUS
begin data;
dimensions ntax=21 nchar=853;
format datatype=dna interleave=yes gap=-;

matrix
PS             GCCCTGGGCAGCCGGCCCCCCTCCAGCTGCCAGCGCACTGTCTGTTCCCC
IW1            RCCMWGGGCAGCCGGCCCCCCTCCAGCTGCCAGCGCACTGTCTGTTCCCC
IW2            GCCMTGGGCAGCCGGCCCCCCTCCAGCTGCCAGCGCACTGTCTGTTCCCC
IW3            GCCCTGGGCAGCCGGCCCCCCTCCAGCTGCCAGCGCACTGTCTGTTCCCC
IW4            GCCCTGGGCAGCCGGCCCCCCTCCAGCTGCCAGCGCACTGTCTGTTCCCC
```

ST3	GCCCTGGGCAGCCGGCCCCCCTCCAGCTGCCAGCGCACTGTCTGTTCCCC
IW5	GCCCTGGGCAGCCGGCCCCCCTMCAGCTGCCAGCGCACTGTCTGTTCCCC
ST2	GCCMTGGGCAGCCGGCCCCCCTCCAGCTGCCAGCGCACTGTCTGTTCCCC
ST1	GCCCTGGGCAGCCGGCCCCCCTCCAGCTGCCAGCGCACTGTCTGTTCCCC
DH1	GCCCTGGGCAGCCGGCCCCCCTCCAGCTGCCAGCGCACTGTCTGTTCCCC
NF2	GCCCTGGGCAGCCGGCCCCCCTCCAGCTGCCAGCGCACTGTCTGTTCCCC
NF1	GCCCTGGGCAGCCGGCCCCCCTCCAGCTGCCAGCGCACTGTCTGTTCCCC
NF3	RCCMWGMGCAGCCGGCSCCCCWCCARCTGCCAGCGCACTGTCTGTTCCCC
NF5	GCCCTGGGCAGCCGGCCCCCCTCCAGCTGCCAGCGCACTGTCTGTTCCCC
ST4	GCCCTGGGCAGCCGGCCCCCCTCCAGCTGCCAGCGCACTGTCTGTTCCCC
ST5	GCCCTGGGCAGCCGGCCCCCCTCCAGCTGCCAGCGCACTGTCTGTTCCCC
NF4	GCCCTGGGCAGCCGGCCCCCCTCCAGCTGCCAGCGCACTGTCTGTTCCCC
DH2	WGTGCTYMCCRATGTCTTGCCTCTGGCGAGCGGTGCGCCATGCGTTCTCC
DH3	WGTGCTYMCCRATGTCTTGCCTCTGGCGAGCGGTGCGCCATGCGTTCTCC
DH4	GSCMWRGGCAGCCGGCCCCCCTCCAGCTGCCAGCGCACTGTCTGTTCCCC
DH5	GSCMWRGGWAGYCGGCCCCCCTCCAGCTGCCAGCGCACTGTCTGTTCCCC

PS	TGGTGCGGCCTGCTGCTGGACACCCGACCCTGGAGGTGTCCTGGACACCC
IW1	TGGTGCGGCCTGCTGCTGGACACCCGACCCTGGAGGTGTCCTGGACACCC
IW2	TGGTGCGGCCTGCTGCTGGACACCCGACCCTGGAGGTGTCCTGGACACCC
IW3	TGGTGCGGCCTGCTGCTGGACACCCGACCCTGGAGGTGTCCTGGACACCC
IW4	TGGTGCGGCCTGCTGCTGGACACCCGACCCTGGAGGTGTCCTGGACACCC
ST3	TGGTGCGGCCTGCTGCTGGACACCCGACCCTGGAGGTGTCCTGGACACCC
IW5	TGGTGCGGCCTGCTGCTGGACACCCGACCCTGGAGGTGTCCTGGACACCC
ST2	TGGTGCGGCCTGCTGCTGGACACCCGACCCTGGAGGTGTCCTGGACACCC
ST1	TGGTGCGGCCTGCTGCTGGACACCCGACCCTGGAGGTGTCCTGGACACCC
DH1	TGGTGCGGCCTGCTGCTGGACACCCGACCCTGGAGGTGTCCTGGACACCC
NF2	TGGTGCGGCCTGCTGCTGGACACCCGACCCTGGAGGTGTCCTGGACACCC
NF1	TGGTGCGGCCTGCTGCTGGACACCCGACCCTGGAGGTGTCCTGGACACCC

NF3	TGGTGCGGCCTGCTGCTGGACACCCGACCCTGGAGGTGTCCTGGACACCC
NF5	TGGTGCGGCCTGCTGCTGGACACCCGACCCTGGAGGTGTCCTGGACACCC
ST4	TGGTGCGGCCTGCTGCTGGACACCCGACCCTGGAGGTGTCCTGGACACCC
ST5	TGGTGCGGCCTGCTGCTGGACACCCGACCCTGGAGGTGTCCTGGACACCC
NF4	TGGTGCGGCCTGCTGCTGGACACCCGACCCTGGAGGTGTCCTGGACACCC
DH2	TGGGGTGGGAGGTGGGTGGGCATGCGTCCCTGGAGGTGTGCTGCACACAC
DH3	TGGGGTGGGAGGTGGGTGGGCATGCGTCCCTGGAGGTGTGCTGCACACAC
DH4	TGGTGCGGCCTGCTGCTGGACACCCGACCCTGGAGGTGTCCTGGACACCC
DH5	TGGTGCGGCCTGCTGCTGGACACCCGACCCTGGAGGTGTCCTGGACACCC

PS	AGGTGAGCGCACCCACCAGGGATGCTGCTTAGCACGCTCCCACTCCTGTC
IW1	AGGTGAGCGCACCCACCAGGGATGCCACTTAGCACGCTCCCACTCCTGTC
IW2	MGGTGAGCSCACCCMCCAGGGATGCCACTTAGCACGCTCCCACTCCTGTC
IW3	AGGTGAGCSCACCCACCAGGGATGCCACTTAGCACGCTCCCACTCCTGTC
IW4	AGGTGAGCGCACCCACCAGGGATGCYRCTTAGCACGCTCCCACTCCTGTC
ST3	AGGTGAGCGCACCCACCAGGGATGCYRCTTAGCACGCTCCCACTCCTGTC
IW5	AGGTGAGCGCACCCACCAGGGATGCCACTTAGCACGCTCCCACTCCTGTC
ST2	AGGTGAGCGCACCCACCAGGGATGCYRCTTAGCACGCTCCCACTCCTGTC
ST1	AGGTGAGCGCACCCACCAGGGATGCCMCTTAKCMCSCTCCCACTCCTGTC
DH1	AGGTGAGCGCACCCACCAGGGATGCCACTTAGCACGCTCCCACTCCTGTC
NF2	AGGTGAGCSCACCCMCCAGGGATGCYRCTTAGCACGCTCCCACTCCTGTC
NF1	AGGTGAGCSCACCCMCCAGGGATGCYRCTTAGCACGCTCCCACTCCTGTC
NF3	AGGTGAGCSCACCCACCAGGGATGCCACTTARCACGCTCCCACTCCTGTC
NF5	AGGTGAGCGCACCCMCCAGGGATGCYRCTTAGCACGCTCCCACTCCTGTC
ST4	AGGTGAGCSCMCCCMCCAGGGATGCYRCTTAGCACSCTCCCACTCCTGTC
ST5	AGGTGAGCSCACCCMCCAGGGATGCCACTTAGCACGCTCCCACTCCTGTC
NF4	AGGTGAGCGCACCCACCAGGGATGCCACTTAGCACGCTCCCACTCCTGTC
DH2	AGGAACGACAGCTCCCCAGGGGCGCCTCTCTGTACGTGCTCACTGGTGGT
DH3	AGGAACGACAGCTCCCCAGGGGCGCCTCTCTGTACGTGCTCACTGGTGGT

DH4	AGGTGAGCSCACCCMCCAGGGATGCCACTTAGCACGCTCCCACTCCTGTC
DH5	AGGTGAGCGCACCCMCCAGGGATGCYRCTTAGCACSCTCCCACTCCTGTC
PS	TCCACAAGGGATCCCGAGAGGGTCCAGACGGGGGCAGAACACTCCTCCTT
IW1	TCCACAAGGGWKVCCGMAGAGGTCCAGACGGGGGCAGAACACTCCTCCTT
IW2	TCCACAAGGGVDCCCGHGAGGGTCCAGACGGGGGCAGAACACTCCTCCTT
IW3	TCCACAAGGGATCCCGHBHBGGTCCAGACGGGGGCAGAACACTCCTCCTT
IW4	TCCACAAGRGATCCCGARAVGDTCCAGACGGGGGCAGAACACTCCTCCTT
ST3	TCCACAAGGSADVVCGAGAAGGTCCAGACGGGGGCAGAACACTCCTCCTT
IW5	TCCACAAGGGATCCCGAGAGGGTCCAGACGGGGGCAGAACACTCCTCCTT
ST2	TCCACAAGGGATCCCGARAGGGTCCAGACGGGGGCARAACACTCCTCCTT
ST1	WCCACAAGGRVHVHCGAGABDKTCCAGACGGGGGCAGAACACTHCTCBTT
DH1	TCCACAAGKGATCCBGDRADDKTCCAGACGGGGGCAGAACACTCCTCCTT
NF2	TCCACAAGGGATCCCGAGAGGGTCCAGACGGGGGCAGAACACTCCTCCTT
NF1	TCCACAAGGGATVCCGWGWVGVTCCAGACGGGGGCAGAACACTCCTCCTT
NF3	TCCACAAGGGATCCCGARAGGGTCCAGACGGGGGCAGAACACTCCTCCTT
NF5	YCCACAAGGGADCHCDAGWVGGTCCAGACGGGGGCAGAACACTCCTCCTT
ST4	TCCACAAGGGATCCCGAGAVGKTCCRGACGGGGGCAGAACACTCCTCCTT
ST5	TCCACAAGGGATHCCGWGATGGTCCAGACGGGGGCAGAACACTCCTCCTT
NF4	TCCACAAGGGATCCCGABADGGTCCAGACGGGGGCAGAACACTCCTCCTT
DH2	GCTCAGGGCCGACCACTTGGGKTCCKKAYKGSSKCRGRRACMTWSTCCTY
DH3	GCTCAGGGCCGACCACTTGGGKTCCKKAYKGSSKCRGRRACMTWSTCCTY
DH4	TCCACAAGRDAWCBYNVGDGGVTCCAGACGGGGGCAGAACACTCCTCCTT
DH5	TCCACAAGGGAHCCCBAGVGVGTCCAGACGGGGGCAGAACACTCCTCCTT
PS	GGGATCGAGAGCACTGGCCAGGCGAGCGGTCCCCTGGGCTGTCTGTGGTA
IW1	GGGATCGAGAGCACTGGCCAGGCGAGCGGTCCCCTGGGCTGTCTGTGGTA
IW2	GGGATCGAGAGCACTGGCCAGGCGAGCGGTCCCCTGGGCTGTCTGTGGTA
IW3	GGGATCGAGAGCACTGGCCAGGCGAGCGGTCCCCTGGGCTGTCTGTGGTA

IW4	GGGATCGAGAGCACTGGCCAGGCGAGCGGTCCCCTGGGCTGTCTGTGGTA
ST3	GGGATCGAGAGCACTGGCCAGGCGAGCGGTCCCCTGGGCTGTCTGTGGTA
IW5	GGGATCGAGAGCACTGGCCAGGCGAGCGGTCCCCTGGGCTGTCTGTGGTA
ST2	GGGATCGAGAGCACTGGCCAGGCGAGCGGTCCCCTGGGCTGTCTGTGGTA
ST1	GGGATCGAGAGCACTBBCCAGDCGAGCDGTCCCCTGGGCTGTCTGTGGTA
DH1	GGGATCGAGAGCACTGGCCAGGCGAGCGGTCCCCTGGGCTGTCTGTGGTA
NF2	GGGATCGAGAGCACTGGCCAGGCGAGCGGTCCCCTGGGCTGTCTGTGGTA
NF1	GGGATCGAGAGCACTGGCCAGGCGAGCGGTCCCCTGGGCTGTCTGTGGTA
NF3	GGGATCGAGAGCACTGGCCAGGCGAGCGGTCCCCTGGGCTGTCTGTGGTA
NF5	GGGATCGAGAGCACTGGCCAGGCGAGCBGTCCCCTGGGCTGTCTGTGGTA
ST4	GGGATCGAGAGCACTGGCCAGGCGAGCGGTCCCCTGGGCTGTCTGTGGTA
ST5	GGGATCGAGAGCACTGGCCAGGCGAGCGGTCCCCTGGGCTGTCTGTGGTA
NF4	GGGATCGAGAGCACTGGCCAGGCGAGCGGTCCCCTGGGCTGTCTGTGGTA
DH2	GRTGYYGWRAKC-CTGTGTAGGMSAKCKSTCYSCTGGGCTGTYTGTGGKA
DH3	GRTGYYGWRAKC-CTGTGTAGGMSAKCKSTCYSCTGGGCTGTYTGTGGKA
DH4	GGGATCGAGAGCACTGGCCAGGCGAGCGGTCCCCTGGGCTGTCTGTGGTA
DH5	GGGATCGAGAGCACTGGCCAGGCGAGCDGTCCCCTGGGCTGTCTGTGGTA

PS	CAAGGAATCTGGGCAAGAGACGGCATCACCGGTGTGCTCTGCAGGGAGGT
IW1	CAAGGAATCTGGGCAAGAGACGGCATCACCGGTGTGCTCTGCAGGGAGGT
IW2	CAAGGAATCTGGGCAAGAGACGGCATCACCGGTGTGCTCTGCAGGGAGGT
IW3	CAAGGAATCTGGGCAAGAGACGGCATCACCGGTGTGCTCTGCAGGGAGGT
IW4	CAAGGAATCTGGGCAAGAGACGGCATCACCGGTGTGCTCTGCAGGGAGGT
ST3	CAAGGAATCTGGGCAAGAGACGGCATCMCCGGTGTGCTCTGCAGGGAGGT
IW5	CAAGGAATCTGGGCAAGAGACGGCATCACCGGTGTGCTCTGCAGGGAGGT
ST2	CAAGGAATCTGGGCAARARACGGCATCACCGGTGTGCTCTGCAGGGAGGT
ST1	CAAGGAATCTGGGCAAGAGACGGCATCACCGGTGTGCTCTGCAGGGAGGT
DH1	CAAGGAATCTGGGCAAGAGACGGCATCACCGGTGTGCTCTGCAGGGAGGT
NF2	CAAGGAATCTGGGCAAGAGACGGCATCMCCGGTGTGCTCTGCAGGGAGGT

NF1	CAAGGAATCTGGGCAAGAGACGGCATCMCCGGTGTGCTCTGCAGGGAGGT
NF3	CAAGGAATCTGGGCAARAGACGGCATCACCGGTGTGCTCTGCAGGGAGGT
NF5	CAAGGAATCTGGGCAAGAGACGGCATCACCGGTGTGCTCTGCAGGGAGGT
ST4	CAAGGAATCTGGGCAAGAGACGGCATCACCGGTGTGCTCTGCAGGGAGGT
ST5	CAAGGAATCTGGGCAAGAGACGGCATCACCGGTGTGCTCTGCAGGGAGGT
NF4	CAAGGAATCTGGGCAAGAGACGGCATCACCGGTGTGCTCTGCAGGGAGGT
DH2	CAASGAATCTGGGCAAGAGACSGCATCMCCGSTGTGCTCTGCAGGGAGGT
DH3	CAASGAATCTGGGCAAGAGACSGCATCMCCGSTGTGCTCTGCAGGGAGGT
DH4	CAAGGAATCTGGGCAAGAGACGGCATCACCGGTGTGCTCTGCAGGGAGGT
DH5	CAAGGAATCTGGGCAAGAGACGGCATCACCGGTGTGCTCTGCAGGGAGGT

PS	TTGGGGACGGCTGCACACAAGTCTCCTCAGGACAAGGGCACACTCCTGAC
IW1	TTGGGGACGGCTGCACACAAGTCTCCTCAGGACAAGGGCACAMTCCTGAC
IW2	TTGGGGACGGCTGCACACAAGTCTCCTCAGGACAAGGGCACAMTCCTGAC
IW3	TTGGGGACGGCTGCACACAAGTCTCCTCAGGACAAGGGCACACTCCTGAC
IW4	TTGGGGACGGCTGCACACAAGTCTCCTCAGGACAAGGGCACAMTCCTGAC
ST3	TTGGGGACGGCTGCACACAAGTCTCCTCAGGACAAGGGCACAMTCCTGAC
IW5	TTGGGGACGGCTGCACACAAGTYTCYTCAGGACAAGGGCACAATMCCRYC
ST2	TTGGGGACGGCTGCMCACAAGTCYCCTCAGGACAAGGGCMCAATCCRYCT
ST1	TTGGGGACGGCTGCACACAAGTCTCCTCAGGACAAGGGCACAMTCCTGAC
DH1	TTGGGGACGGCTGCACACAAGTCTCCTCAGGACAAGGGCACAMTCCTGAC
NF2	TTGGGGACGGCTGCACACAAGTCTCCTCAGGACAAGGGCACAACMKSRCW
NF1	TTGGGGACGGCTGCACACAAGTCTCCTCAGGACAAGGGCACAMTCCTGAC
NF3	TTGGGGACGGCTGCACACAAGTCTCCTCAGGACAAGGGCACAATMMCKRS
NF5	TTGGGGACGGCTGCACACAAGTCTCCTCAGGACAAGGGCACAMTCCTGAC
ST4	TTGGGGACGGCTGCACACAAGTCTCCTCAGGACAAGGGCACAMTCCTGAC
ST5	TTGGGGACGGCTGCACACAAGTCTCCTCAGGACAAGGGCACAMTCCTGAC
NF4	TTGGGGACGGCTGCACACAAGTCTCCTCAGGACAAGGGCACAMTCCTGAC
DH2	TTGGGGACSGCTGCMCMCAASTCTCCTCARGACAAGGGCACACTCCTGAC

DH3	TTGGGGACSGCTGCMCMCAASTCTCCTCARGACAAGGGCACACTCCTGAC
DH4	TTGGGGACGGCTGCACACAAGTCTCCTCAGGACAAGGGCACAMTCCTGAC
DH5	TTGGGGACGGCTGCACACAAGTCTCCTCAGGACAAGGGCACAMHCCTGAC

PS	GCCCTCTGTGGTCGGGGTCCTCTTCAGGCCGCTCCTTGGCACATAGACGT
IW1	SCCCTCTGTGGTCGGGGTCCTCTTCARGCCGCTCCTTGGCMCATAKACSS
IW2	GCCCTCTGTGGTCGGGGTCCTCTTCAGGCCGCTCCTTGGCACATAGACGC
IW3	GCCCTCTGTGGTSGGGGTCCTCTTCAGGCCGCTCCTTGGCACATAGACGC
IW4	GCCCTCTGTGGTCGGGGTCCTCTTCAGGCCGCTCCTTGGCACATARACGY
ST3	SCCCTCTGTGGTCGGGGTCCTCTTCAGGCCGCTCCTTGGCACATARACGY
IW5	WCTSTMGWSGGACGGGGCCYCCCCCCGGCCCTGGCTTGGCAGAGACACGG
ST2	GSTGTMGWGGGACGGGGCCTCCCCCCGGCCCTGGCTTGGCAGAGACACGG
ST1	GCCCTCTGTGGTSGRKSTCCTCTTCHGGCCGCTCCTTGGCACATAGACGC
DH1	SCCCTCTGTGGTCGGGGTCCTCTTCAGGCCGCTCCTTGGCACATAGACGC
NF2	GCTGTSAYSGGTSGRKSTSYWCTTCAGGCCGCTCCTTGGCACATAGACGY
NF1	SCCCTCTGTGGTCGGGGTCCTCTTCAGGCCGCTCCTTGGCACATAGACGY
NF3	TRRWGTMGYSMGCWSGAKCWYACTWMGGCCGCTCCTTGGCACATAGACGC
NF5	GCCCTCTGTGGTCGGGGTCCTCTTCAGGCCGCTCCTTGGCACATAGACGY
ST4	GCCCTCTGTGGTCGGGGTCCTCTTCAGGCCGCTCCTTGGCACATAGACGY
ST5	GCCCTCTGTGGTCGGGGTCCTCTTCAGGCCGCTCCTTGGCACATAGACGC
NF4	GCCCTCTGTGGTCGGGGTCCTCTTCAGGCCGCTCCTTGGCACATAGACGC
DH2	SCCCTCTGTGGTSGRGGTCCTCTTCAGGCCGCTCCTTGGCACATAGACGT
DH3	SCCCTCTGTGGTSGRGGTCCTCTTCAGGCCGCTCCTTGGCACATAGACGT
DH4	GCCCTCTGTGGHSKGGGWCYWCTTCRKGCCGCTCCTTGRBMCATAGACGC
DH5	GCCCWCTGTGGTCGGGGTVCTCTTCAGGCCGCTCCTTGGCACATARACGY

PS	GGGGTCTGTGTCCTCACGTCTCTCAAGACGCTCCCCTGTGGCGCCAGGCC
IW1	GGGGYCTGTGTCCTCACSYCYCTCAAGACGCTCCCCTGTGGCGCCAGGCC
IW2	GGGGTCTGTGTCCTCACGTCTCYCAAGACGCTCCCCTGTGGCGCCAGGCC

IW3	GGGGTCTGTGTCCTCACGTCTCTCAAGACGCTCCCCTGTGGCGCCAGGCC
IW4	GGGGTCTGTGTCYTCACGTCTCTCAAGACGCTCCCCTGTGGCGCCAGGCC
ST3	GGGGTCTGTGTCCTCACGTCYCTCAAGACGCTCCCCTGTGGCGCCARGCC
IW5	TGGACGTGGGTGCTGGAATCTGCWGWMSACGWTCYCTGTGRCRMCARGCC
ST2	TGGACGTGGGTGCTGGAATCTGCWAAGACGCTCCCCTGTGRCGCCAGGCC
ST1	GGGGTCTGTGTCCTCACGTCTCHCAAGACRCTYSSCTMTGRYKCCAMKYS
DH1	GGGGTCTGTGTCCTCACGTCTCTCAAGACGCTCCCCTGTGGCGCCAGGCC
NF2	GGGGTCTGTGTCCTCACGTCTCTCAAGACGCTCCCCTGTGGCGCCAGGCC
NF1	GGGGTCTGTGTCCTCACGTCTCTCAAGACGCTCCCCTGTGGCGCCAGGCC
NF3	GGGRTCYGTGWCCTCACGTCTCTCAAGACGCTCCCCTGTGGCGCCAGGCC
NF5	GGGGTCTGTGTCCTCACGTCTYTCAAGACGCTCCCCTGTGGCGCCAGGCC
ST4	GGGGTCTGTGTCCTCACGTCTCTCAAGACGCTCCCCTGTGGCGCCAGGCC
ST5	GGGGTCTGTGTCCTCACGTCTCTCAAGACGCTCCCCTGTGGCGCCAGGCC
NF4	GGGGTCTGTGTCCTCACGTCTCTCAAGACGCTCCCCTGTGGCGCCAGGCC
DH2	GGGGTCTGTGTCCTCACGTCTCTCAAGACGCTCCCCTGTGGCGCCAGGCC
DH3	GGGGTCTGTGTCCTCACGTCTCTCAAGACGCTCCCCTGTGGCGCCAGGCC
DH4	GGGGTCTGTGTCCTCACGTCTCTCAAGACGCTCCCCTGTGGCGCCAGGCC
DH5	GGGGTCTGTGTCCTCACGTCTCTHAAGACGCTCCCCTGTGGCGCCAGGCC

PS	AGGAAGCTCTCCGGCCCCAGAGGGCCTGTGCCCCTGAGTCTGCCTGTTGC
IW1	AGGAAGCTCTCCGGCCCCAGAGGGCCTGTGCCCCTGAGTCTGCCTGTTGC
IW2	AGGAAGCTCTCCGGCCCCAGAGGGCCTGTGCCCCTGAGTCTGCCTGTTGC
IW3	AGGAAGCTCTCCGGCCCCAGAGGGCCTGTGCCCCTGAGTCTGCCTGTTGC
IW4	AGGAAGCTCTCCGGCCCCAGAGGGCCTGTGCCCCTGAGTCTGCCTGTTGC
ST3	ARGAAGCTCTCCGGCCCCAGAGGGCCTGTGCCCCTGAGTCTGCCTGTTGC
IW5	AGGAAGCTCTCCGGCCCCARAGGGCCTGTGCCCCTGAGTCTGCCTGTTGC
ST2	AGGAAGCTCTCCGGCCCCAGAGGGCCTGTGCCCCTGAGTCTGCCTGTTGC
ST1	ARGAWGCTMTCCGG-CCMAGAGGGCCTGTGCCCCTGAGTCTGCCTGTTGC
DH1	AGGAAGCTCTCCGGCCCCAGAGGGCCTGTGCCCCTGAGTCTGCCTGTTGC

NF2	AGGAAGCTCTCCGGCCCCAGAGGGCCTGTGCCCCTGAGTCTGCCTGTTGC
NF1	AGGAAGCTCTCCGGCCCCAGAGGGCCTGTGCCCCTGAGTCTGCCTGTTGC
NF3	AGGAAGCTCTCCGGBCCCAGAGGGCCTGTGCCCCTGAGTCTGCCTBTTGC
NF5	AGGAAGCTCTCCGGCCCCAGAGGGCCTGTGCCCCTGAGTCTGCCTGTTGC
ST4	AGGAAGCTCTCCGGCCCCAGAGGGCCTGTGCCCCTGAGTCTGCCTGTTGC
ST5	AGGAAGCTCTCCGGCCCCAGAGGGCCTGTGCCCCTGAGTCTGCCTGTTGC
NF4	AGGAAGCTCTCCGGCCCCAGAGGGCCTGTGCCCCTGAGTCTGCCTGTTGC
DH2	AGGAAGCTCTCCGGCCCCAGAGGGCCTGTGCCCCTGAGTCTGCCTGTTGC
DH3	AGGAAGCTCTCCGGCCCCAGAGGGCCTGTGCCCCTGAGTCTGCCTGTTGC
DH4	AGGAAGCTCTCCSGCCCCAGAGGGCCTGTGCCCCTGAGTCYGCCTGTTGC
DH5	AGGAAGCTCTCCGGCCCCAGAGGGCCTGTGCCCCTGAGTCTGCCTGTTGC

PS	GTGTCCACTGTCGTGTCGGGTGGTGCAGAGCTCACCCGTTCAGCCGGGGC
IW1	GTGTCCACTGTCGTGTCGGGTGGTGCAGAGCTCACCCGTTCAGCCGGGGC
IW2	GTGTCCACTGTCGTGTCGGGTGGTGCAGAGCTCACCCGTTCAGCCGGGGC
IW3	GTGTCCACTGTCGTGTCGGGTGGTGCAGAGCTCACCCGTTCAGCCGGGGC
IW4	GTGTCCACTGTCGTGTCGGGTGGTGCAGAGCTCACCCGTTCAGCCGGGGC
ST3	GTGTCCACTGTCGTGTCGGGTGGTGCAGAGCTCACCCGTTCAGCCGGGGC
IW5	GTGTCCACTGTCSTGTCGGGTGGTGCAGAGCTCACCCGTTCASCCGGGGC
ST2	GTGTCCACTGTCGTGTCGGGTGGTGCAGAGCTCACCCGTTCAGCCGGGGC
ST1	GTGTCCACTGTCSTGTCGGGTGGTGCAGAGCTCACCCGTTCAGCCSGGGC
DH1	GTGTCCACTGTCGTGTCGGGTGGTGCAGAGCTCACCCGTTCAGCCGGGGC
NF2	GTGTCCACTGTCGTGTCGGGTGGTGCAGAGCTCACCCGTTCAGCCGGGGC
NF1	GTGTCCACTGTCGTGTCGGGTGGTGCAGAGCTCACCCGTTCAGCCGGGGC
NF3	GTGTCCACTGTCGTGTCGGGTGGTGCAGAGCTCACCCGTTCAGCCGGGGC
NF5	GTGTCCACTGTCGTGTCGGGTGGTGCAGAGCTCACCCGTTCAGCCGGGGC
ST4	GTGTCCACTGTCGTGTCGGGTGGTGCAGAGCTCACCCGTTCAGCCGGGGC
ST5	GTGTCCACTGTCGTGTCGGGTGGTGCAGAGCTCACCCGTTCAGCCGGGGC
NF4	GTGTCCACTGTCGTGTCGGGTGGTGCAGAGCTCACCCGTTCAGCCGGGGC

DH2	GTGTCCACTGTCSTGTCSGGTGGTGCAGAGCTCACCCGTTCAGCCGGGGC
DH3	GTGTCCACTGTCSTGTCSGGTGGTGCAGAGCTCACCCGTTCAGCCGGGGC
DH4	GTGTCCACTGTCSTGTCGGGTGGTGCAGAGCTCACCCGTTCAGCCGGGGC
DH5	GTGTCCACTGTCSTGTCGGGTGGTGCAGAGCTCACCCGTTCAGCCGGGGC

PS	TCCCACGGGGCTCAGGGCTCTGCCTCTGTCCTTGAAACCTGTTGCCTCCA
IW1	TCCCACGGGGCTCAGGGCTCTGCCTCTGTCCTTGAAACCTGTTGCCTCCA
IW2	TCCCACGGGGCTCAGGGCTCTGCCTCTGTCCTTGAAACCTGTTGCCTCCA
IW3	TCCCACGGGGCTCAGGGCTCTGCCTCTGTCCTTGAAACCTGTTGCCTCCA
IW4	TCCCACGGGGCTCAGGGCTCTGCCTCTGTCCTTGAAACCTGTTGCCTCCA
ST3	TCCCACGGGGCTCAGGGCTCTGCCTCTGTCCTTGAAACCTGTTGCCTCCA
IW5	TCCCACGGGGCTCAGGGCTCTGCCTCTGTCCTTGAAACCTGTTGCCTCCA
ST2	WCCCACGGGGCTCAGGGCTCTGCCTCTGTCCTTGAAACCTGTTGCCTCCA
ST1	TCCCACGGGGCTCARGGCTCTGCCTCTGTCCTTGAAACCTGTTGCCYCCA
DH1	TCCCACGGGGCTCAGGGCTCTGCCTCTGTCCTTGAAACCTGTTGCCTCCA
NF2	TCCCACGGGGCTCAGGGCTCTGCCTCTGTCCTTGAAACCTGTTGCCTCCA
NF1	TCCCACGGGGCTCAGGGCTCTGCCTCTGTCCTTGAAACCTGTTGCCTCCA
NF3	TCCCACGGGGCTCAGGGCTCTGCCTCTGTCCTTGAAACCTGTTGCCTCCA
NF5	TCCCACGGGGCTCAGGGCTCTGCCTCTGTCCTTGAAACCTGTTGCCTCCA
ST4	TCCCACGGGGCTCAGGGCTCTGCCTCTGTCCTTGAAACCTGTTGCCTCCA
ST5	TCCCACGGGGCTCAGGGCTCTGCCTCTGTCCTTGAAACCTGTTGCCTCCA
NF4	TCCCACGGGGCTCAGGGCTCTGCCTCTGTCCTTGAAACCTGTTGCCTCCA
DH2	TCCCACGGGGCTCAGGGCTCTGCCTCTGTCCTTGAAACCTGTTGCCTCCA
DH3	TCCCACGGGGCTCAGGGCTCTGCCTCTGTCCTTGAAACCTGTTGCCTCCA
DH4	TCCCACGGGGCTCAGGGCTCTGCCTCTGTCCTTGAAACCTGTTGCCYCCA
DH5	TCCCACGGGGCTCAGGGCTCTGCCTCTGTCCTTGAAACCTGTTGCCTCCA

PS	ACCACGCACCTCTGCTCTCATGGGCGGCCTGCCTGACGTCTCTGGAAACA
IW1	ACCACGCACCTCTGCTCTCATGGGCGGCCTGCCTGACGTCTCTGGAAACA

IW2	ACCACGCACCTCTGCTCTCATGGGCGGCCTGCCTGACGTCTCTGGAAACA
IW3	ACCACGCACCTCTGCTCTCATGGGCGGCCTGCCTGACGTCTCTGGAAACA
IW4	ACCACGCACCTCTGCTCTCATGGGCGGCCTGCCTGACGTCTCTGGAAACA
ST3	ACCACGCACCTCTGCTCTCATGGGCGGCCTGCCTGACGTCTCTGGAAACA
IW5	ACCACGCACCTCTGCTCTCATGGGCGGCCTGCCTGACGTCTCTGGAAACA
ST2	ACCACGCACCTCTGCTCTCATGGGCGGCCTGCCTGACGTCTCTGGAAACA
ST1	ACCACGCACCTCTGCTCTCATGGGCGGCCTGCCTGACGTCTCTGGAAACA
DH1	ACCACGCACCTCTGCTCTCATGGGCGGCCTGCCTGACGTCTCTGGAAACA
NF2	ACCACGCACCTCTGCTCTCATGGGCGGCCTGCCTGACGTCTCTGGAAACA
NF1	ACCACGCACCTCTGCTCTCATGGGCGGCCTGCCTGACGTCTCTGGAAACA
NF3	ACCACSCAHHTCTGCTCTCATGGGCGGCCTGCCTGACGTCTCTGGAAACA
NF5	ACCACGCAMCTCTGCTCTCATGGGCGGCCTGCCTGACGTCTCTGGAAACA
ST4	ACCACGCAMCTCTGCTCTCATGGGCGGCCTGCCTGACGTCTCTGGAAACA
ST5	ACCACGCAMCTCTGCTCTCATGGGCGGCCTGCCTGACGTCTCTGGAAACA
NF4	ACCACGCAMCTCTGCTCTCATGGGCGGCCTGCCTGACGTCTCTGGAAACA
DH2	ACCACGCACCTCTGCTCTCATGGGCGGCCTGCCTGACGTCTCTGGAAACA
DH3	ACCACGCACCTCTGCTCTCATGGGCGGCCTGCCTGACGTCTCTGGAAACA
DH4	ACCMCSCACCTCTGCTCTCATGGGCGGCCTGCCTGACGTCTCTGGAAACA
DH5	ACCACGCACCTCTGCTCTCATGGGCGGCCTGCCTGACGTCTCTGGAAACA

PS	TGCTCAGAGTGTAGCACGAACGTGCCCACACGCCACACACGGTGAGATTG
IW1	TGCTCAGAGTGTAGCACGAACGTGCCCACACGCCACACACGGTGAGATTG
IW2	TGCTCAGAGTGTAGCACGAACGTGCCCACACGCCACACACGGTGAGATTG
IW3	TGCTCAGAGTGTAGCACGAACGTGCCCACACGCCACACACGGTGAGATTG
IW4	TGCTCAGAGTGTAGCACGAACGTGCCCACACGCCACACACGGTGAGATTG
ST3	TGCTCAGAGTGTAGCACGAACGTGCCCACACGCCACACACGGTGAGATTG
IW5	TGCTCAGAGTGTAGCACGAACGTGCCCACACGCCACACACGGTGAGATTG
ST2	TGCTCAGAGTGTAGCACGAACGTGCCCACACGCCACACACGGTGAGATTG
ST1	TGCTCAGARTGTASCACGAACGTGCCCACACGCCACACACGGTGAGATTG

DH1	TGCTCAGAGTGTAGCACGAACGTGCCCACMCSCCACACACGGTGAGATTG
NF2	TGCTCAGAGTGTAGCACGAACGTGCCCACMCSCCACACACGGTGAGATTG
NF1	TGCTCAGAGTGTAGCACGAACGTGCCCACMCSCCACACACGGTGAGATTG
NF3	TGCTCARAGTGTAGCACGAACGTGCCCMCACSCCACACACGGTGAGATTG
NF5	TGCTCAGAGTGTAGCACGAACGTGCCCACMCSCCACACACGGTGAGATTG
ST4	TGCTCAGAGTGTAGCACGAACGTGCCCACACSCCACACACGGTGAGATTG
ST5	TGCTCAGAGTGTAGCACGAACGTGCCCACACSCCACACACGGTGAGATTG
NF4	TGCTCAGAGTGTAGCACGAACGTGCCCACMCSCCACACACGGTGAGATTG
DH2	TGCTCAGAGTGTAGCACGAACGTGCCCACMCSCCACACACGGTGAGATTG
DH3	TGCTCAGAGTGTAGCACGAACGTGCCCACMCSCCACACACGGTGAGATTG
DH4	TGCTCAGAGTGTAGCACGAACGTGCCCACMCSCCACACACGGTGAGATTG
DH5	TGCTCAGAGTGTAGCACGAACGTGCCCACACSCCACACACGGTGAGATTG

PS	GAGGTGGTGGACGCTCACCGGCAGCTCTGTAAGCTCCTCAGTTCAAGCAT
IW1	GAGGTGGTGGACGCTCAACAGCAGCTCTGTAAGCTCCTCAGTTCAAGCAT
IW2	GAGGTGGTGGACGCTCAAGGGCAGCTCTGTAAGCTCCTCAGTTCAAGCAT
IW3	GAGGTGGTGGACGCTCAACGGCAGCTCTGTAAGCTCMTCAGTTCAAGCAT
IW4	GAGGTGGTGGACGCTCAACGGCAGCTCTGT-AGCTCCTCASTTCAAGCAT
ST3	GAGGTGGTGGACGCTCAACKGCAGCTCTGT-AGCTCCTCAGTTCAAGCAT
IW5	GAGGTGGTGGACSCTCAAARGCAGCTCTGTAAGCTCCTCASTTCAAGCAT
ST2	GAGGTGGTGGACGCTCAGCGGCAGCTCTGT-AGCTCCTCAGTTCAAGCAT
ST1	GAGGTGGTGGACGCTCAAGGGCAGCTCTGT-AGCTCCTCAGTTCAAGCAT
DH1	GAGGTGGTGGACGCYCAGCGGCAGCTCTGTAAGCTMMTCAGTTCAAGCAT
NF2	GAGGTGGTGGACGCYCAACGGCAGCTCTGTAAGCTMMTCAGTTCAAGCAT
NF1	GAGGTGGTGGACGCYCAACGGCAGCTCTGTAAGCTMMTCAGTTCAAGCAT
NF3	GAGGTGGTGGACGCTCAAGGAGCAGCTCYSTASCTMMTCAGTTCAAGCAT
NF5	GAGGTGGTGGACGCTCCCWGGCAGCTCTGTAAGCTCCTCAGTTCAAGCAT
ST4	GAGGTGGTGGACGCTCACCGGCAGCTCYGT-ASCTMCTCAGTTCARGCWY
ST5	GAGGTGGTGGACGCTCAACGGCAGCTCTGT-AGCTCCTCAGTTCAAGCAT

NF4	GAGGTGGTGGACGCTCAKYGGCAGCTCTGTAAGCTCMTCAGTTCAAGCAT
DH2	GAGGTGGTGGACGCTCAACGGCAGCTCTGT-AGCTCCTCAGTTCAAGCAT
DH3	GAGGTGGTGGACGCTCAACGGCAGCTCTGT-AGCTCCTCAGTTCAAGCAT
DH4	GAGGTGGTGGACGCTCACTGRCRGCTCTSWAAGCTMMTCAGTTCAAGCAT
DH5	GAGGTGGTGGACGCTCASKGGCRGCTCTGTAAGCTCCTCAGTTCAAGCAT

PS	GTGCAGCCCTATGACGGCAGGTGCTGCATGCTACGGGCTTCTCCACCCAC
IW1	GKGCAGCCCTATGACGGCAGGTGCTGCATGCTACGGGCTTCTCCACCCAC
IW2	GTGCAGCCCTATGACGGCAGGTGCTGCATGCTACGGGCTTCTCCACCCAC
IW3	GTGCAGCCCTATGACGGCAGGTGCTGCATGCTACGGGCTTCTCCACCCAC
IW4	GTGCAGCCCTATGACGGCAGGTGCTGCATGCTACSGGCTTCTCCACCCAC
ST3	GTGCAGCCCTATGACGGCAGGTGCTGCATGCTACGGGCTTCTCCACCCAC
IW5	GTGCAGCCCTATGACGGCAGGTGCTGCATGCTACGGGCTTCTCCACCCAC
ST2	GTGCAGCCCTATGACGGCAGGTGCTGCATGCTACGGGCTTCTCCACCCAC
ST1	GTGCAGCCCTATGACGGCAGGTGCTGCATGCTACGGGCTTCTCCACCCAC
DH1	GTGCAGCCCTATGACGGCAGGTGCTGCATGCTACGGGCTTCTCCACCCAC
NF2	GTGCAGCCCTATGACGGCAGGTGCTGCATGCTACGGGCTTCTCCACCCAC
NF1	GTGCAGCCCTATGACGGCAGGTGCTGCATGCTACGGGCTTCTCCACCCAC
NF3	GTGCAGCCCTATGACGGCAGGTGCTGCATGCTACGGGCTTCTCCACCCAC
NF5	GTGCAGCCCTATGACGGCAGGTGCTGCATGCTACGGGCTTCTCCACCCAC
ST4	GTGCAGCCCTATGACGGCAGGTGCTGCATGCTACGGGCTTCYCCACCCAC
ST5	GTGCAGCCCTATGACGGCAGGTGCTGCATGCTACGGGCTTCYCCACCCAC
NF4	GTGCAGCCCTATGACGGCAGGTGCTGCATGCTACGGGCTTCTCCACCCAC
DH2	GTGCAGCCCTATGACGGCAGGTGCTGCATGCTACGGGCTTCTCCACCCAC
DH3	GTGCAGCCCTATGACGGCAGGTGCTGCATGCTACGGGCTTCTCCACCCAC
DH4	GTGCAGCCCTATGACGGCAGGTGCTGCATGCTACGGGCTTCTCCACCCAC
DH5	GTGCAGCCCTATGACGGCAGGTGCTGCATGCTACGGGCTTCYCCACCCAC

PS	GGGCATGTGTGTCAGTAGTTTCCATGTGGATGTCGAGGCACGTGGGAGGC

IW1	GGGCATGTGTGTCAGTAGTTTCCATGTGGATGTCSAGGCACGTSGGAGGC
IW2	GGGCATGTGTGTCAGTAGTTTCCATGTGGATGTCSAGGCACGTGGGAGGC
IW3	GGGCATGTGTGTCAGTAGTTTCCATGTGGATGTCSAGGCACSTGGGAGGC
IW4	GGGCATGTGTGTCAGTAGTTTCCATGTGGATGTCSAGGCACSTGGGAGGC
ST3	GGGCATGTGTGTCAGTAGTTTCCATGTGGATGTCGAGGCACGTGGGAGGC
IW5	GGGCATGTGTGTCAGTAGTTTCCATGTGGATGTCSAGGCMCGTGGGAGGC
ST2	GGGCATGTGTGTCAGTAGTTTCCATGTGGATGTCSAGGCACGTGGGAGGC
ST1	GGGCATGTGTGTCAGTAGTTTCCATGTGGATGTCSAGGCMCSTGGGAGGC
DH1	GGGCATGTGTGTCAGTAGTTTCCATGTGGATGTCGAGGCACGTGGGAGGC
NF2	GGGCATGTGTGTCAGTAGTTTCCATGTGGATGTCGAGGCACGTGGGAGGC
NF1	GGGCATGTGTGTCAGTAGTTTCCATGTGGATGTCGAGGCACGTGGGAGGC
NF3	GGGCATGTGTGTCAGTAGTTTCCATGTGGATGTCSAGGCACGTGGGAGGC
NF5	GGGCATGTGTGTCAGTAGTTTCCATGTGGATGTCGAGGCACGTGGGAGGC
ST4	GGGCATGTGTGTCMGTARTTTCCATGTGGATGTCSAGGCACSTGGGAGGC
ST5	GGGCATGTGTGTCAGTAGTTTCCATGTGGATGTCSAGGCACSTGGGAGGC
NF4	GGGCATGTGTGTCAGTAGTTTCCATGTGGATGTCSAGGCMCGTGGGAGGC
DH2	GGGCATGTGTGTCAGTAGTTTCCATGTGGATGTCSAGGCMCGTGGGAGGC
DH3	GGGCATGTGTGTCAGTAGTTTCCATGTGGATGTCSAGGCMCGTGGGAGGC
DH4	GGGCATGTGTGTCAGTAGTTTCCATGTGGATGTCSAGGCACGTGGGAGGC
DH5	GGGCATGTGTGTCAGTAGTTTCCATGTGGATGTCSAGGCMCGTGGGAGGC

PS	TCT
IW1	TCT
IW2	TCT
IW3	TCT
IW4	TCY
ST3	TCT
IW5	TCY
ST2	TCT

```
ST1           TCY
DH1           TCT
NF2           TCT
NF1           TCT
NF3           TCT
NF5           TCT
ST4           TCT
ST5           TCT
NF4           TCY
DH2           TCT
DH3           TCT
DH4           TCT
DH5           TCY
;
end;
```
